# Serotonin hormonally regulates lacrimal gland secretory function via the serotonin type 3a receptor

**DOI:** 10.1038/s41598-017-06022-4

**Published:** 2017-07-31

**Authors:** Toshihiro Imada, Shigeru Nakamura, Ryuji Hisamura, Yusuke Izuta, Kai Jin, Masataka Ito, Naoki Kitamura, Kenji F. Tanaka, Masaru Mimura, Izumi Shibuya, Kazuo Tsubota

**Affiliations:** 1 Department of Ophthalmology, Keio University School of Medicine, 35 Shinanomachi, Shinjyuku-ku, Tokyo 160-8582 Japan; 2 Department of Developmental Anatomy and Regenerative Biology, National Defense Medical College, 3-2 Namiki, Tokorozawa, Saitama 359-8513 Japan; 3 Department of Veterinary Physiology, Faculty of Agriculture, Tottori University, 4-101, Koyama-cho Minami, Tottori, 680-8553 Japan; 4 Department of Neuropsychiatry, Keio University School of Medicine, 35 Shinanomachi, Shinjyuku-ku, Tokyo 160-8582 Japan

## Abstract

Tears are extracellular fluid secreted from the lacrimal gland (LG). Tears consist of a dynamic tri-layered film composed of secretions from the LG, Meibomian gland, and conjunctival goblet cells. The LG secretes the aqueous component of the tear, the Meibomian gland secretes the lipid component, and conjunctival goblet cells secrete mucin. The regulation of LG activity via the autonomic nervous system has been recognized as fundamental to maintaining aqueous tear flow. Here, we describe the role of a hormone, peripheral serotonin, in tear secretion. We found that blood serotonin concentration, changed by feeding a diet deprived of the serotonin precursor tryptophan, correlated with tear secretion, and that a sustained decrease in serotonin resulted in LG atrophy and autophagy. The combination of a decrease in serotonin with the interruption of autonomic neural stimuli to the LG preceded these alterations. Furthermore, we found that the serotonin type 3a receptor expressed in LG acinar cells is involved in tear secretion via intracellular calcium mobilization. Our findings demonstrate that hormonal regulation by serotonin, in cooperation with the autonomic nervous system, regulates tear secretion.

## Introduction

Physiological functions of peripheral organs are regulated by direct innervation by the central nervous system and a wide variety of hormones released from the different glands or tissues from which they are synthesized^1, 2^. For example, the autonomic nervous system and circulating hormones released from endocrine organs regulate cardiac and pancreatic secretory functions, respectively. Heart rate is increased by activation of the sympathetic nervous system and secretion of adrenalin from the adrenal medulla^3^ and is decreased by activation of the parasympathetic nervous system and secretion of calcitonin gene-related peptides from the thyroid^4^. Pancreatic fluid secretion form the exocrine pancreas gland is predominantly parasympathetic and involves various hormones, of which gastrointestinal secretin and somatostatin play a role in its stimulation and suppression, respectively^5, 6^.

Serotonin (5-hydroxytryptamine, 5-HT) is a biogenic monoamine synthesized from dietary L-tryptophan (Trp), and is mainly produced by peripheral organs^7^. 5-HT is not only a central neurotransmitter but also acts as a hormone to regulate various peripheral extraneuronal functions, including vasoconstriction^8^ and gastrointestinal motility^9^. These hormonal actions are controlled through fourteen subtypes of 5-HT receptors (5-HTR) that are classified into seven families^10^.

Aqueous tear fluid secretion from the lacrimal gland (LG) is necessary for ocular surface health and serves to maintain clear vision by creating a smooth, reflective surface and providing oxygen to the avascular corneal epithelium^11^. Both the sympathetic and parasympathetic components of the autonomic nervous system innervate the LG, and the parasympathetic system plays an essential role in the regulation of LG tear secretory function^12^. Parasympathetic stimulation by acetylcholine (ACh) induces tear secretion by activating M3 muscarinic Ach receptors (M3R) located in the basolateral membrane of LG acinar cells^13^. Although a variety of hormonal receptors such as epidermal growth factor receptor^14^, androgen receptor^15^, adiponectin receptor^16^, and angiotensin receptor^17^ have been proposed to exist in the LG, the contribution of individual receptors to the regulation of tear secretion is not fully understood.

Insufficient aqueous tear secretion leads to abnormalities in the ocular surface^11^, ocular discomfort^18^, and functional visual acuity^19^, resulting in dry-eye syndrome^11^. The etiology of this syndrome appears to be multifactorial^20^, but little attention has been directed to the potential involvement of an imbalance between the autonomous nervous system and hormonal factors. Here, we investigated the role of peripheral 5-HT in tear secretion. The relationship between blood 5-HT concentration and LG secretory function was evaluated by feeding a Trp-free diet to mice, an intervention that constantly lowers blood 5-HT level without toxic effects^21^. In addition, we identified and analyzed the role of 5-HTR in LG secretory function. We found that in addition to autonomic innervation, peripheral hormonal 5-HT also plays a role in tear secretion, and that LG 5-HT3aR mediates these activities.

## Results

### Effects of a Trp-free diet on tear secretion

**Figure 1:**
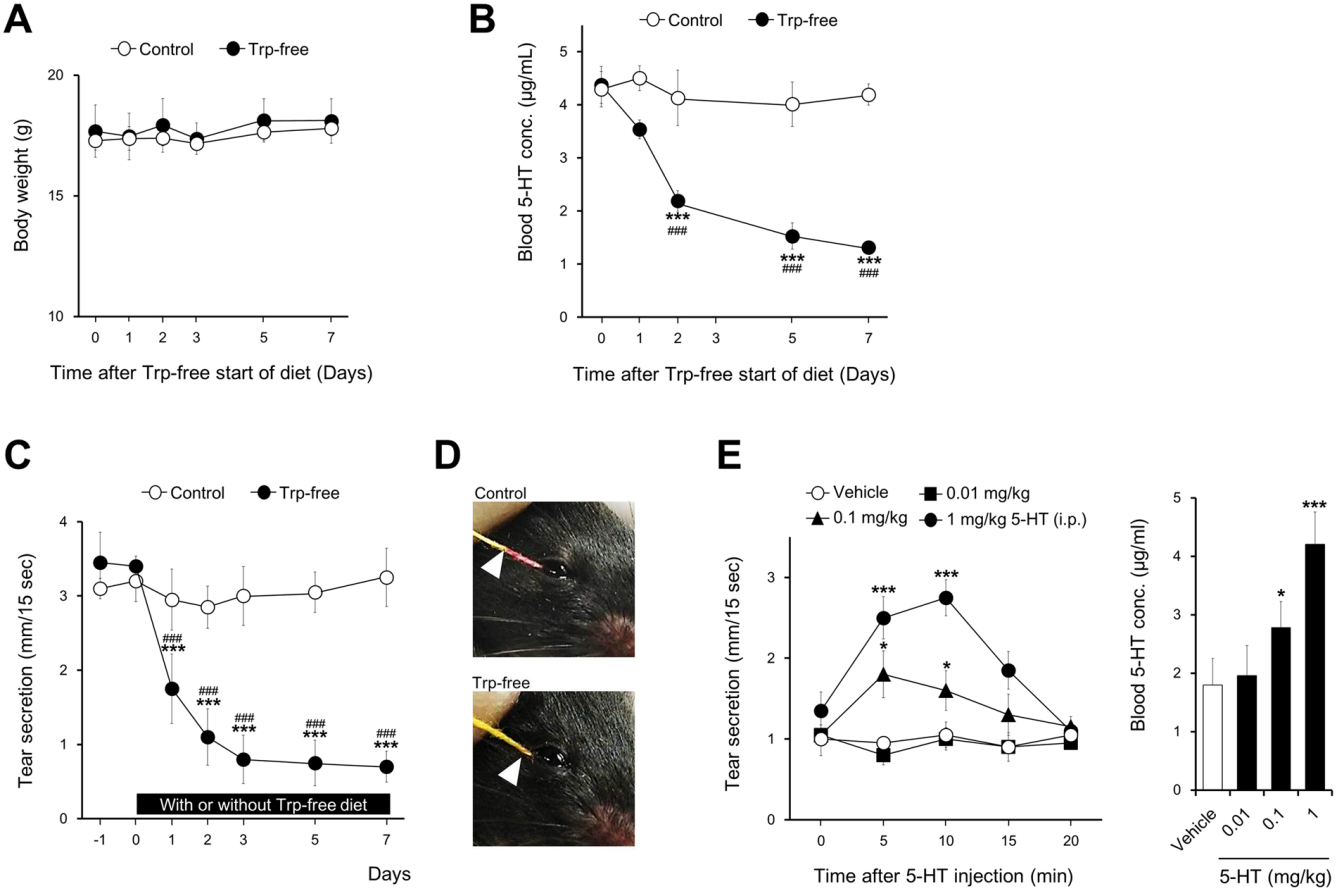
Effects of a Trp-free diet on tear secretion. Mice were given a standard diet (control group) or Trp-free diet (Trp-free group) for 7 days after the acclimatization period. Changes in blood 5-HT concentration (**A**) and body weight (**B**). (**C**) Changes in tear secretion. Each measurement was performed during 7 days of feeding with a standard diet or Trp-free diet. Open and solid symbols show the control group and Trp-free group, respectively (**A**–**C**). (**D**) Representative photographs of tear secretion patterns measured with a cotton thread at day 7 after changing to a Trp-free diet. The arrow indicates the wetted length due to tear secretion. (**E**) Stimulated tear secretion by 5-HT. 5-HT was injected 2 days after changing to a Trp-free diet. The line chart shows the changes in tear secretion after injection with vehicle (saline, open circles) and 5-HT at the dosage of 0.01 mg/kg (solid squares), 0.1 mg/kg (solid triangles), and 1 mg/kg (solid circles). The bar chart shows the blood 5-HT concentration 10 minutes after injection with vehicle (open column) or 5-HT (solid columns) at each dosage corresponding to the line chart. All data represent the mean ± SD, n = 6 mice. *P < 0.05, ***P < 0.001 versus control (**B**,**C**). ^#^ *P* < 0.001 versus day 0 (**B**,**C**), *P < 0.05, ***P < 0.001 versus vehicle (**E**).

We investigated the physiological role of blood 5-HT in tear secretion. Mouse blood 5-HT concentration was decreased with a diet deficient in Trp, a 5-HT precursor^22^. In the control group, there was no change in body weight and blood 5-HT concentration compared with initial values (Fig. 1A and B, open circles). Throughout the experiment, body weight was not modified after changing from a standard diet to a Trp-free diet (Fig. 1A, closed circles). Blood 5-HT concentration gradually decreased from day 1 after changing to a Trp-free diet, and was decreased by approximately 70% from day 7 compared with the initial value before changing diet (Fig. 1B, closed circles). A significant decrease in blood 5-HT concentration was observed from days 2 to 7 compared with the value before changing to a Trp-free diet (day 0) and the control group.

After changing to a Trp-free diet, tear secretion gradually decreased and plateaued, at approximately 20% of the initial value, between days 3 and 7 (Fig. 1C). A significant decrease was observed on days 1 to 7 compared with the initial value at day 0. Tear secretion was significantly less on days 1 to 7 in the Trp-free group compared with that in the control group. Figure 1D shows representative photographs of tear secretion patterns measured by a modified Schirmer test. The wetted length on the thread showed by a red discolored area was shorter in the Trp-free diet group (days 7) than in the control group.

There was no significant difference in tear secretion associated with sexual dimorphism after changing to a Trp-free diet (Supplementary Figure 1).

Decreased tear secretion induced by feeding a Trp-free diet was gradually recovered after reverse switch from Trp-free to standard diet. A significant increase in tear secretion was observed from days 3 to 7 compared with before switching to standard diet (day 0). On day 7, the tear secretion was almost returned to those before feeding a Trp-free diet (Supplementary Figure 2). Reduction in tear secretion was sustained for 30 days as long as mice were fed a Trp-free diet (data not shown).

To investigate whether 5-HT directly activates LG secretory function, we evaluated the change in tear secretion after systemic injection of 5-HT. Serotonin was intraperitoneally (i.p.) injected after 2 days of feeding a Trp-free diet. This time point was chosen to accurately measure the effect of exogenously injected 5-HT on tear secretion, as a significant reduction of blood 5-HT was achieved with minimal feeding duration of a Trp-free diet, which minimized the functional effect on this diet on the LG. Intraperitoneal injections of 5-HT dose-dependently stimulated tear secretion under the condition that blood 5-HT was reduced by feeding a Trp-free diet (Fig. 1E). The stimulatory effect was elicited from 5 to 10 minutes after 5-HT injection, and was significantly higher at the dosages of 0.1 and 1 mg/kg 5-HT compared with that when injecting the vehicle. Similarly to the time course of the recovery of tear secretion, 5-HT injections dose-dependently resulted in a recovery of the blood 5-HT concentration. These results suggest that changes in blood 5-HT concentration correlate with tear secretion.

### Decreased blood 5-HT induces LG atrophy

The LG and Meibomian gland (MG) are major glandular organs for tear production. The mouse LG is located on the surface of the subcutaneous masseter muscle inferior to the ear, whereas the MG is spread in the subcutaneous region of the upper and lower eyelid. Conjunctival goblet cells, sparse among non-mucus secreting conjunctival epithelium, are the primary source for the mucous component of tear film.

**Figure 2:**
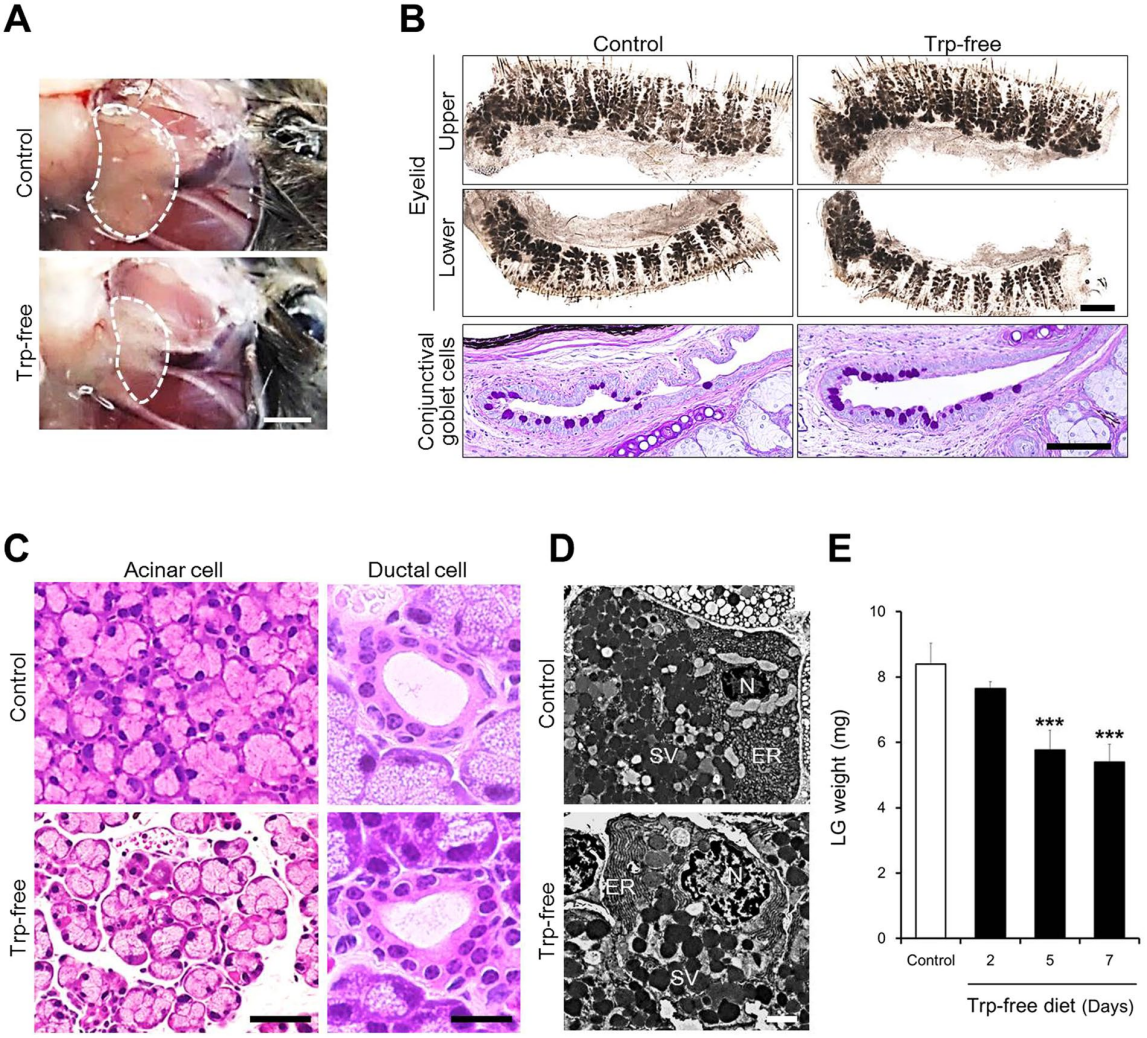
Decrease in blood 5-HT concentration induces lacrimal gland atrophy. (**A**) Effect of Trp-free diet on the LG (upper: control, lower Trp-free diet group). The dotted line indicates LG (scale bar: 2 mm). (**B**) Effect of Trp-free diet on MG morphology and conjunctival goblet cell distribution. MG morphology was assessed by whole-mount eyelid meibography (scale bar: 100 µm). Conjunctival goblet cells were stained by PAS (scale bar: 100 µm). Number of conjunctival goblet cells was 32 in the control and 36 in the Trp-free group. Each panel shows the control group (left) and Trp-free diet group (right). (**C**) Histopathological changes in LG acinar cells and ductal cells. Scale bar is 50 µm (acinar cells) and 10 µm (ductal cells). (**D**) Typical transmission electron microscopy (TEM) images of LG acinar cells (scale bar: 1 µm). The nucleus, secretory vesicles, and endoplasmic reticulum are shown as N, SV, and ER, respectively. Each panel (C,D) shows the control group (upper) and Trp-free group (lower). Photographs show at day 7 after changing to a Trp-free diet (**A**–**D**). (**E**) Changes in LG weight during the 7 days of Trp-free diet feeding. All data represent the mean ± SD, n = 5 LGs. ***P < 0.001 versus control (**E**).

Changes in LG, MG morphology and conjunctival goblet cell distribution after 7 days of feeding a Trp-free diet are shown in Fig. 2A and B. In the Trp-free group, the LG was atrophied compared with that of the control group (Fig. 2A). In contrast to the LG, whole-mount eyelid meibography, a technique used to assess morphological characteristics of the MG, did not reveal morphological differences in MG morphology, gland number, nor ductal branching with lobular development in the Trp-free group compared with that in the control group (Fig. 2B). Periodic acid-Schiff (PAS) staining for mucin-producing goblet cells of the bulbar and tarsal conjunctiva demonstrated that conjunctival goblet cell density and mucus content in cells were maintained at the same level as that in the Trp-free group compared with that in the control group.

Since decreased tear secretion accompanied by LG atrophy were characteristic changes due to feeding a Trp-free diet, we performed a histopathological examination of LG stained with hematoxylin and eosin (HE) to evaluate their morphological characteristics (Fig. 2C). In the Trp-free diet group, the size of acinar cells was reduced and the epithelial thickness and lumen of the duct was unchanged as compared to that in the control group. The LG lobule structure was maintained in both the control and Trp-free diet groups. Transmission electron microscopy (TEM) observation revealed that secretory vesicles and organelles, especially the endoplasmic reticulum, were decreased in the atrophied LG in animals from the Trp-free diet group (Fig. 2D).

In the Trp-free group, atrophic changes in the LG gradually progressed from day 2 after changing the diet to a Trp-free diet, and the LG weight on day 5 became approximately 60% of that of the control group (Fig. 2E). No significant difference in the LG weight was observed between day 5 and day 7 after changing to a Trp-free diet.

Atrophy is characterized by a decrease in tissue mass due to either a decrease in cell volume and/or number caused by increases in protein degradation^23^. Autophagy is a catabolic process for the degradation and recycling of proteins that is essential for maintenance of cellular homeostasis^24^. Excess activation of autophagy is reported to be involved in organ atrophy^25^. To further understand the role of blood 5-HT in LG function, we investigated the involvement of autophagy in atrophied LG that was accompanied by a decrease in tear secretion by feeding a Trp-free diet.

**Figure 3:**
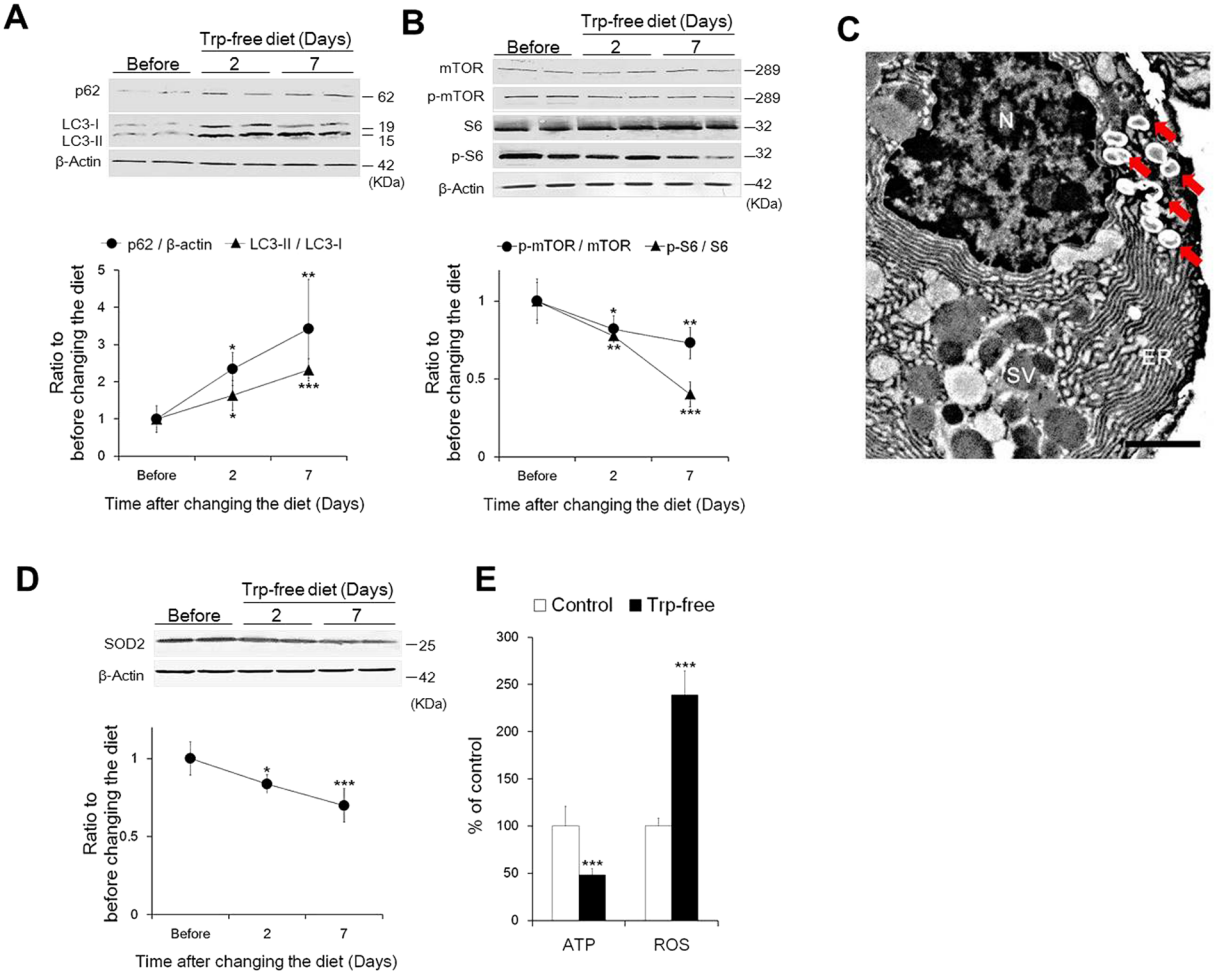
Lacrimal gland atrophy due to decrease in blood 5-HT concentration is induced by activation of autophagy. (**A**) Changes in expression levels of p62 and LC3-II in the LG. (**B**) Changes in the phosphorylation levels of mTOR and S6 in the LG. (**C**) The high magnification TEM image of autophagosomes (red arrows) found in LG acinar cells of the Trp-free diet group. Photograph from day 7 after changing to a Trp-free diet. The nucleus, secretory vesicles, and endoplasmic reticulum are shown as N, SV, and ER, respectively. (**D**) Changes in expression levels of SOD2 in the LG. Data (**A**–**D**) show the representative western blot images and quantitative analysis (line chart) before changing the diet, and 2 days and 7 days after changing to a Trp-free diet. Solid circles and triangles indicate the ratios of p62/β-actin and LC3-II/LC3-I (**A**), and p-mTOR/mTOR and p-S6/S6 (**B**), respectively. (**E**) Alterations in ATP content and ROS generation in the LG at day 7 after changing to a Trp-free diet. Data show the percentage of the value in the Trp-free group relative to that in controls for ATP content and ROS generation. Open and solid columns show the control group and Trp-free group, respectively. All data represent the mean ± SD, n = 5 LGs. *P < 0.05, **P < 0.01, ***P < 0.001 versus before (**A**,**B**,**D**), ***P < 0.001 versus control (**E**).

To evaluate the level of autophagy during Trp-free diet feeding, we analyzed the autophagy-related protein p62, microtubule-associated protein 1 light chain 3 (LC3) and mammalian target of rapamycin (mTOR) pathway, widely used markers of mammalian autophagy^26, 27^. Expression levels of p62 and LC3-II, an indicator of the accumulation of autophagosomes^27^, gradually increased (Fig. 3A). Conversely, the phosphorylation levels of mTOR and its downstream effector ribosomal protein S6 (S6) kinase gradually decreased after changing to a Trp-free diet (Fig. 3B). Significance was observed in each protein at days 2 and 7 compared with initial values from before feeding a Trp-free diet (Fig. 3A and B). On TEM analysis, large cytoplasmic inclusions, membrane-bound vacuoles that characterize the activation of autophagy, were observed in LG acinar cells (Fig. 3C).

These results suggest that the activation of autophagy was involved in the LG atrophy induced by a decrease in blood 5-HT concentration.

Autophagy is triggered by various stimuli, including cellular energy deprivation^28^ and oxidative stress^29^. The expression levels of manganese-SOD (SOD2) and mitochondrial antioxidant isoenzyme^30^, were gradually decreased after changing to a Trp-free diet, and were significantly lower at days 2 and 7 compared with values before feeding a Trp-free diet (Fig. 3D). ATP content in the LG, an indicator of cellular energy status, was significantly lower after 7 days of feeding a Trp-free diet compared with a standard diet. Reactive oxygen species (ROS) generation from the LG, indicating a breakdown of cellular antioxidant defense, was significantly higher compared with that in the standard diet group (Fig. 3E).

The weights of nine peripheral organs were determined and reduction rates were calculated (Supplementary Figure 3). Significance was observed in the liver and submandibular gland in addition to in the LG. The reduction rate of the LG was significantly higher than that of the liver and submandibular gland.

### 5-HT3R mediates tear secretion and LG morphology

5-HT acts through a family of receptors with fourteen subtypes^10^. To determine which 5-HTR subtype was involved in the regulation of tear secretion and LG morphology, we evaluated the effect of several 5-HTR antagonists on tear secretion and LG weight at day 7 by sustained subcutaneous infusion of each antagonist. Among the ubiquitous 5-HTRs, we selected antagonists against 5-HT1aR, 5-HT2aR, 5-HT3R, and 5-HT7R, since their signaling pathways are mediated through second messengers and effector proteins such as phospholipase C, intracellular Ca^2+^, and cyclic AMP^31, 32^.

**Figure 4:**
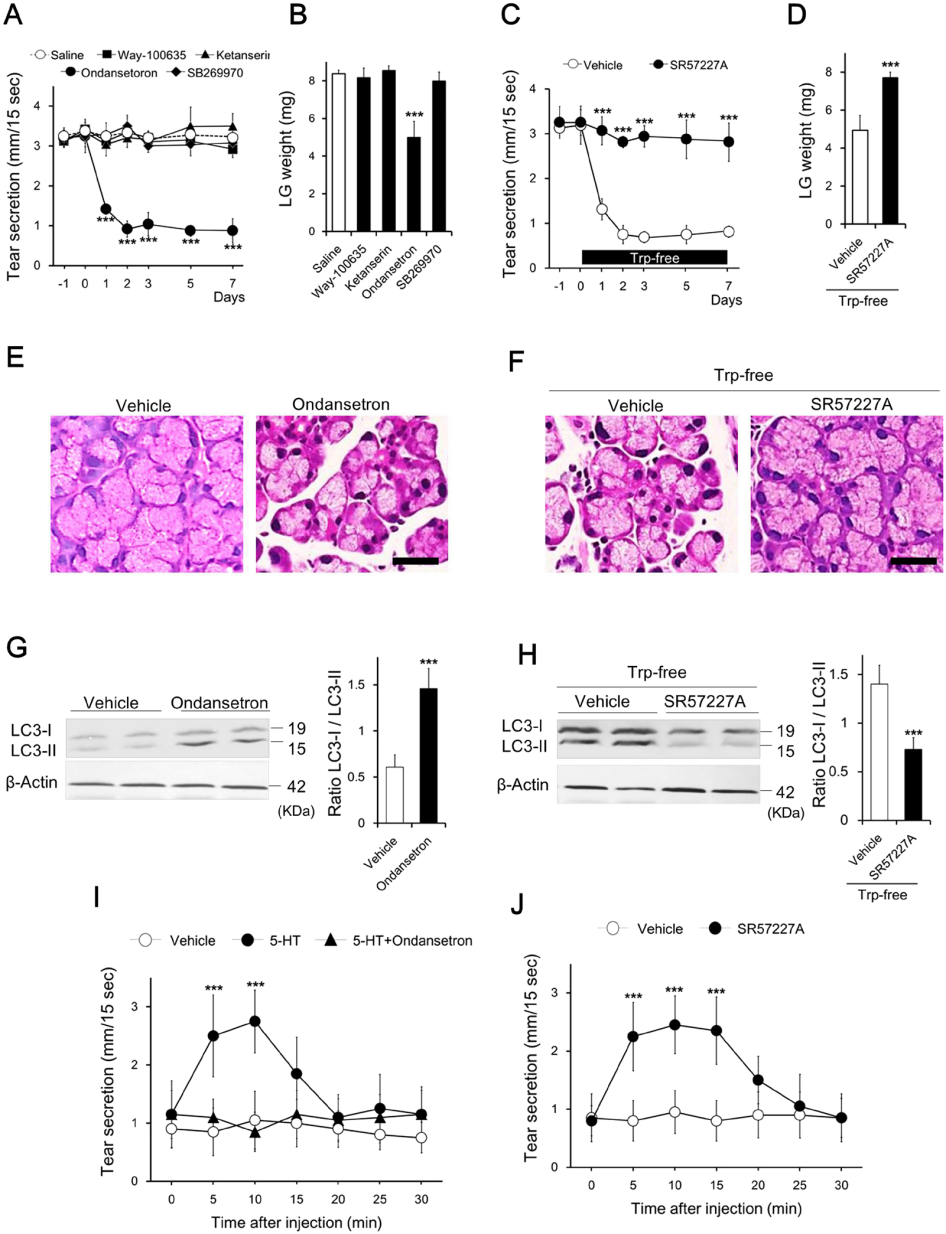
5-HT3R mediates tear secretion and lacrimal gland morphology. (**A**) Changes in tear secretion and (**B**) LG weight by continuous infusion of 5-HTR antagonists (n = 5 mice). Mice were infused with saline (vehicle) or each 5-HT3R antagonist for 7 days via an osmotic pump. Data show the changing ratio of each antagonist to saline with tear secretion (open column) and LG weight (solid column) at day 7 after infusion. Way-100635, ketanserin, ondansetron, and SB269970 were used as antagonists for 5-HT1aR, 5-HT2aR, 5-HT3R, and 5-HT7, respectively. (**C**) Effect of an agonist for 5-HT3R (SR57227A) on tear secretion under a condition of decreased blood 5-HT. SR57227A or vehicle (saline) was infused 1 day before changing to a Trp-free diet (n = 5 mice). (**D**) LG weight after SR57227A infusion (n = 10 LG). Histopathological changes in LG by infusion of (**E**) ondansetron or (**F**) SR57227A (scale bar is 10 µm). Changes in expression of LC3-II by infusion of (**G**) ondansetron or (**H**) SR57227A (n = 5 LG). Representative western blot image and quantitative analysis of the western blot (bar chart). (**I**) Effect of ondansetron on stimulated tear secretion by 5-HT. Ondansetron was simultaneous injected with 5-HT. Open circle, closed circle, and closed triangle indicate injection of vehicle, 5-HT, and 5-HT with ondansetron, respectively. (**J**) Stimulated tear secretion by SR57227A. Open circle and closed circle indicate injection of vehicle and SR5727A, respectively. Each drug was injected 2 days after changing to a Trp-free diet (**I**,**J** n = 5 mice). Data represent the 7 days after injection of a 5-HTR antagonist (**B**,**E**,**G**) and after changing to a Trp-free diet (**D**,**F**,**H**). Open and solid symbols show the infusion of vehicle and ondansetron or SR57227A, respectively (**A**–**D**,**G**,**H**). All data represent the mean ± SD. ***P < 0.001 versus saline (**A**,**B**), vehicle (**C**,**D**,**G**,**H**), or 0 min (**I**,**J**).

Tear secretion decreased and plateaued, at approximately 30% of the initial value, during subcutaneous infusion of ondansetron, a 5-HT3R antagonist. Tear secretion was significantly less on days 1 to 7 in the ondansetron-infused group compared to the saline-infused group; however, the other antagonists (5-HT1aR antagonist Way-100635, 5-HT2aR antagonist ketanserin, and 5-HT7R antagonist SB269970) did not change tear secretion during the experimental period (Fig. 4A). In accordance with changes in tear secretion, LG weight was significantly decreased by ondansetron treatment only among the 5-HTR antagonists, and the value at days 7 was decreased by 50% of that of saline-infused animals (Fig. 4B).

To further verify the involvement of 5-HT3R on LG function, we evaluated the effect of sustained systemic infusion of a 5-HT3R agonist on tear secretion and LG morphology under conditions where 5-HT was decreased by a Trp-free diet. With the infusion of SR57227A, a 5-HT3R agonist, tear secretion did not decrease throughout the period after changing to a Trp-free diet (Fig. 4C), and a decrease in LG weight was not observed (Fig. 4D). Pathological examination showed that ondansetron infusion reduced the size of LG acinar cells, and this alteration corresponded to that observed after changing to a Trp-free diet (Fig. 4E). The pathological change caused by a Trp-free diet was almost completely reversed with SR57227A infusion (Fig. 4F). Furthermore, the morphological status of LG ductal cells was unchanged in all the treatment groups (Supplementary Figure 8A).

The effects of a 5-HT3R agonist and antagonist on the LG autophagic response were also evaluated. Expression of LC3-II was increased by ondansetron infusion (Fig. 4G) and was maintained at normal levels by SR57227A infusion after changing to a Trp-free diet (Fig. 4H). The changes in other autophagy-related markers by ondansetron infusion corresponded to those observed after changing to a Trp-free diet (Supplementary Figure 4).

The inhibitory effect of a 5-HT3R antagonist against stimulated tear secretion with a 5-HT injection and the stimulatory effect of a 5-HT3R agonist injection were confirmed under conditions in which blood 5-HT concentration was reduced by 2 days of feeding a Trp-free diet. Injection of ondansetron inhibited the elevation of tear secretion stimulated by 5-HT (Fig. 4I), and the injection of SR57227A transiently increased tear secretion to the same level as that stimulated by 5-HT (Fig. 4J). We also confirmed that injection of 5-HT and SR57227A stimulated tear secretion in mice with a blood 5-HT concentration under the normal level (Supplementary Figure 5).

### 5-HT elevates intracellular calcium ion concentration ([Ca^2+^]i) through 5-HT3aR in LG acinar cells

An increase in [Ca^2+^]i plays an important role in tear secretion from the LG^12^. 5-HT3R stimulation raises [Ca^2+^]i by extracellular Ca^2+^ entry through the activation of a ligand gate ion channel located in the cell membrane^31^. Since we observed that 5-HT3R may play a role in LG secretory function, we further studied the involvement of 5-HT3R in [Ca^2+^]i mobilization in fluorescent Ca^2+^ indicator (Fura2)-loaded LG acinar cells isolated from normal rat.

**Figure 5:**
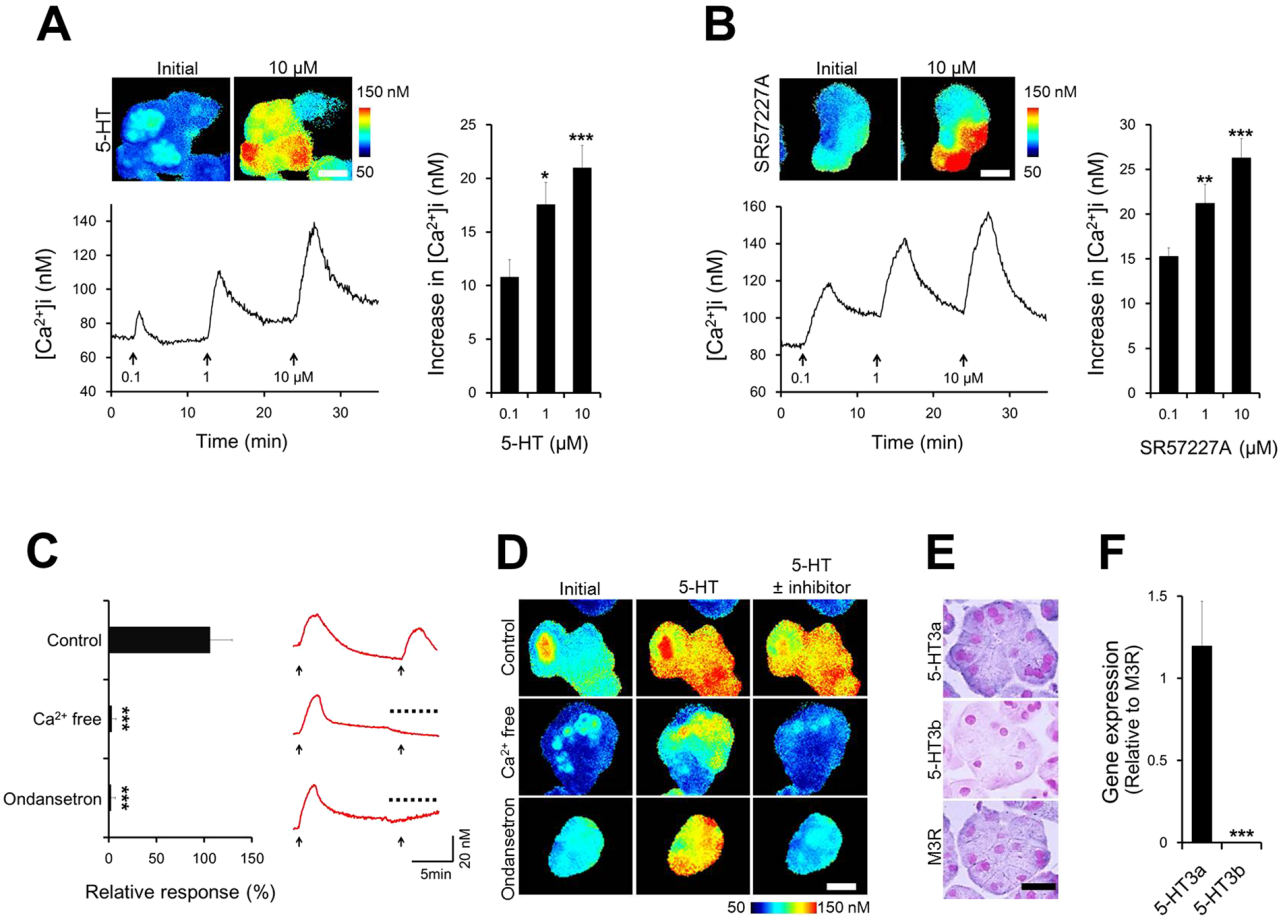
5-HT mobilizes [Ca^2+^]i in lacrimal gland acinar cells via 5-HT3aR. Intracellular calcium mobilization was measured in fura-2 loaded LG acinar cells isolated from normal rat. Changes in [Ca^2+^]i in LG acinar cells by stimulation with (**A**) 5-HT and (**B**) SR57227A. The upper panel shows pseudo-colored images of [Ca^2+^]i before stimulation (left) and after stimulation with 5-HT or SR57227A at a dosage of 10 µM (right) (scale bar, 50 µm). The lower trace shows the typical response to each stimulus. The arrow indicates the time at which 5-HT or SR57227A was applied to the cells. The bar chart shows the summarized data of amplitudes of [Ca^2+^]i responses (**A**: n = 43 acini, B: n = 49 acini). (**C**) Inhibitory effects of ondansetron and a Ca^2+^-free condition on 5-HT-induced [Ca^2+^]i mobilization (n = 11–25 acini). Representative responses obtained under each condition (red trace). The arrow indicates the time at which 5-HT was applied to the cells at dosages of 0.1, 1, and 10 µM, respectively. The dotted line over the trace indicates the presence of an antagonist or a Ca^2+^-free condition. Relative responses were calculated as a percentage of the decrease in the 5-HT-induced [Ca^2+^]i response with the application of a Ca^2+^-free condition or ondansetron relative to the stimulation induced with 5-HT alone in each acinus. Pseudo-colored images of [Ca^2+^]i are shown in (**D**); the scale bar is 50 µm. Gene expression analysis of the 5-HT3R subtype detected by (**E**) ISH (counterstain: nuclear fast red, scale bar: 10 µm) and (**F**) determined by qPCR (n = 6 LG). mRNA expression of the 5-HT3R subtype was determined in normal rat LG. ISHs are shown for the indicated 5-HT3R and M3R probes (**E**). Each panel shows LG acinar cells. All data represent the mean ± SD. *P < 0.05, **P < 0.01, ***P < 0.001 versus 0.1 µM (**A**,**B**) and versus 5-HT3a (**F**).

Stimulation with 5-HT dose-dependently increased [Ca^2+^]i in LG acinar cells at a range from 0.1 to 10 µM (Fig. 5A). An increase in [Ca^2+^]i was also induced by SR57227A stimulation, and the responsiveness of [Ca^2+^]i to SR57227A was the same as that to 5-HT (Fig. 5B). The [Ca^2+^]i elevation by 5-HT was inhibited by extracellular Ca^2+^ removal and pretreatment with ondansetron (Fig. 5C and D).

Tear fluid is a mixture of water from transepithelial flow and excreted intracellular protein released by exocytosis from LG acinar cells triggered by [Ca^2+^]i elevation. To confirm whether 5-HT activates LG exocytosis via 5-HT3R along with [Ca^2+^]i elevation, we measured the amount of total protein release by 5-HT.stimulation from LG acinar cells using isolated normal rat LG. In this measurement, we measured total protein, as an index of exocytotic release of protein from LG acinar cells based on previous studies^33–36^. Stimulation with 5-HT dose-dependently increased the total proteins released from LG acinar cells, and the release of total secretory proteins was completely inhibited by ondansetron (Supplementary Figure 6).

5-HT3R includes two subtypes, 3a and 3b^31^. *In situ* hybridization (ISH) and quantitative PCR in LG were performed to verify the expression pattern of each 5-HT3R subtype. 5-HT3aR mRNA was observed in LG acinar cells, but 5-HT3bR mRNA was not detected (Fig. 5E). Corresponding to ISH results, real-time PCR analysis showed that the expression level of 5-HT3aR was higher than that of 5-HT3bR (Fig. 5F). The 5-HT3aR expression level was the same as that of M3R.

We determined the mRNA expression pattern of the 5-HTR subtypes in the LG isolated from normal mice to verify that the involvement of 5-HT3R in LG [Ca^2+^]i mobilization found in rats was applicable to that in mice (Supplementary Figure 7). 5-HT3aR mRNA was observed in mouse LG acinar cells and other 5-HTR subtypes, but 5-HT3bR was not detected. The expression pattern of 5-HT3R in mouse LG was identical to that in rat LG.

### Interaction between parasympathetic stimulation and 5-HT

Parasympathetic innervation plays a prominent role in tear secretion^37^. We subsequently investigated the effect of the interaction between parasympathetic stimulation and 5-HT on LG secretory function. LG secretory functions were evaluated at day 1 after combined LG parasympathetic post-ganglionic denervation (PGD) and feeding of a Trp-free diet for 4 days.

**Figure 6:**
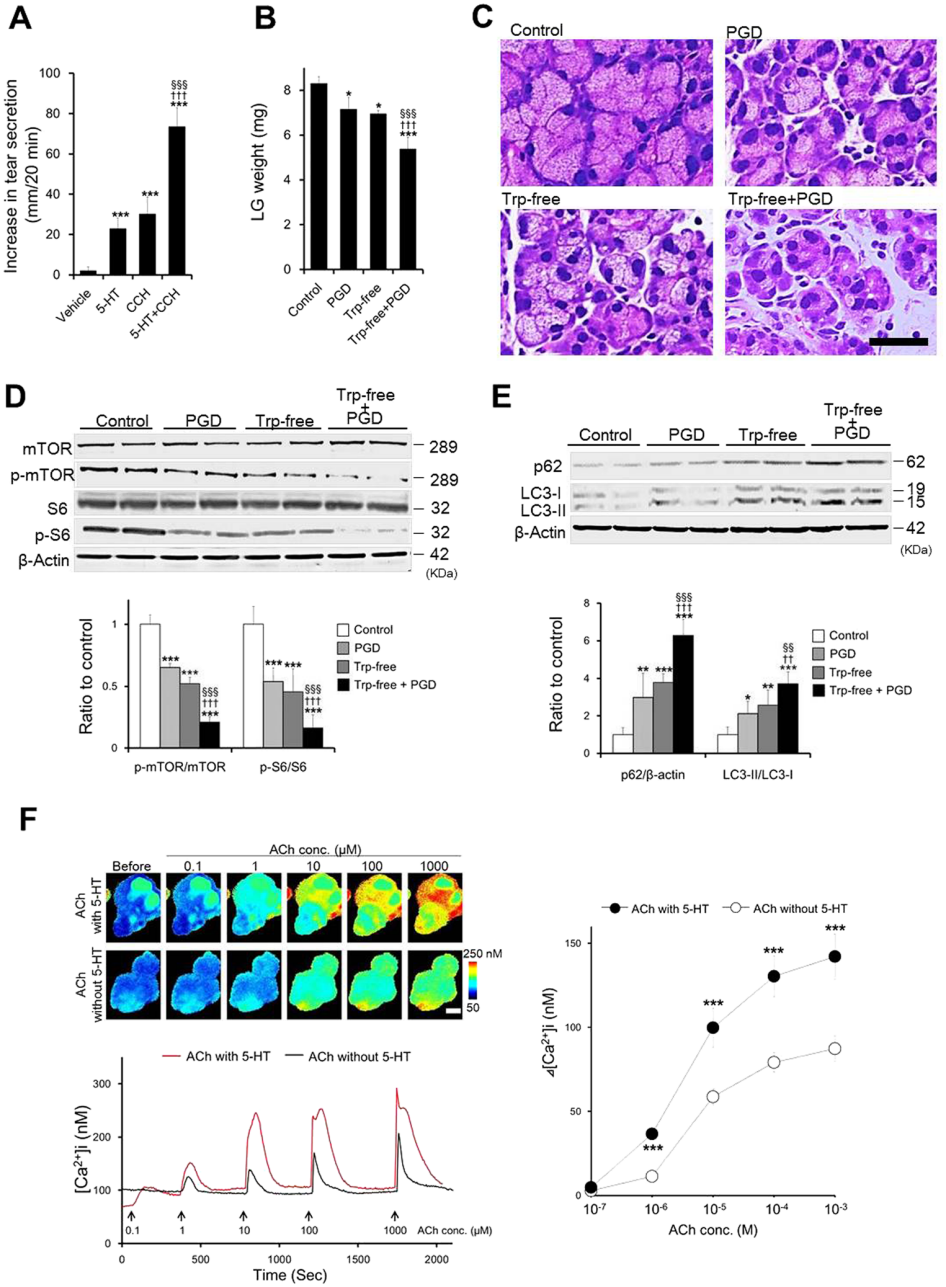
Interaction between parasympathetic stimulation and 5-HT. (**A**) Stimulated tear secretion by 5-HT, CCH, or simultaneous 5-HT with CCH under a condition whereby both parasympathetic and 5-HT stimuli were decreased. Each drug was injected after 1 day of combined PGD and 3 days of Trp-free feeding (n = 5 mice). Sum of tear secretion for 20 minutes after each injection. (**B**) Change in LG weight (n = 6–8 LG) and (**C**) morphology of the LG (scale bar is 10 µm). (**D**) Change in the phosphorylation levels of mTOR and S6 (n = 6 LG). (**D**) Change in expression levels of p62 and LC3-II (n = 6 LGs). Change in expression level of each protein (**D**,**E**) was analyzed by western blot at day 1 of combined PGD and 4 days of Trp-free feeding. Representative western blot image and quantitative analysis of western blot (bar chart). The LG was analyzed at day 1 after PGD (PGD), at day 4 after feeding a Trp-free diet (Trp-free), at day 1 after combination of PGD and 4 days of Trp-free feeding (Trp-free + PGD) (**B**–**E**). (**F**) Interaction between ACh and 5-HT on [Ca^2+^]i elevation in LG acinar cells (n = 33–35 acini). Acinar cells isolated from normal rat LG were stimulated by ACh at dosages between 0.1 µM to 1 mM with or without 5-HT at a constant dosage (1 µM); each stimulation was performed for 1 minute. The upper panel shows pseudocolored images of [Ca^2+^]i (scale bar, 50 µm). The lower trace shows the typical response to each stimulus. The arrow indicates the time at which ACh with or without 5-HT was applied to the cells. The line chart show the summarized amplitudes of [Ca^2+^]i responses. All data represent the mean ± SD, ***P < 0.001 versus vehicle, ^†††^ *P* < 0.001 versus 5-HT, ^§§§^ *P* < 0.001 versus CCH (**A**), *P < 0.05, ***P < 0.001 versus control, ^§§§^ *P* < 0.01 versus PGD, ^§§§^ *P* < 0.001 versus Trp-free (**B**,**D**,**E**), ***P < 0.001 versus without 5-HT (**F**).

Intraperitoneal injections of 5-HT or the parasympathetic stimulant carbachol (CCH) stimulated tear secretion compared with saline injection under conditions whereby both parasympathetic and 5-HT stimuli of LG were decreased (Fig. 6A). Stimulated tear secretion by combined injection of 5-HT and CCH was significant, approximately 3-fold and 2.4-hold higher than that by individual injections of either 5-HT or CCH, respectively.

Compared with the normal condition, a significant decrease in LG weight was observed at day 1 after PGD and 4 days after feeding a Trp-free diet (Trp-free) (Fig. 6B). Under a condition of PGD combined with a Trp-free diet (Trp-free + PGD), LG weight was significantly decreased to approximately 77% and 75% of that in PGD and a Trp-free diet, respectively.

Histopathological evaluation of the LG showed the size of acinar cells under a condition of PGD combined with a Trp-free diet was reduced compared with that of PGD and a Trp-free diet (Fig. 6C). The morphological status of LG ductal cells was unchanged in all the treatment groups (Supplementary Figure 8B).

Consistent with changes in LG weight and morphology, activation of autophagy-related proteins phosphor-mTOR, phosphor-S6, p62, and LC3-II significantly proceeded toward autophagy under a condition of PGD combined with a Trp-free diet compared with PGD or a Trp-free diet (Fig. 6D and E).

The simultaneous effects of 5-HTR and M3R on [Ca^2+^]i were evaluated in Fura-2–loaded acinar cells isolated from normal rat. A dose-dependent [Ca^2+^]i increase in response to Ach was observed with or without 5-HT (Fig. 6F). The [Ca^2+^]i increase in response to ACh with 5-HT was approximately 2-fold higher than that without 5-HT. A significant elevation was observed from 1 to 1,000 µM compared with ACh without 5-HT.

These observations suggest that 5-HT interacts cooperatively with parasympathetic stimuli to control LG secretory function.

## Discussion

Parasympathetic innervation plays a central role in tear fluid secretion from the LG^12^. The principal observation of the present study was that LG secretory functions correlated with levels of blood 5-HT. Furthermore, parasympathetic stimulation and blood 5-HT cooperatively interacted to modulate LG secretory functions. This suggests that both parasympathetic innervation and hormonal 5-HT provide essential signals for maintaining LG secretory functions.

There are more than seven major types and fourteen subtypes of 5-HTR, which are ubiquitously distributed in various tissues such as the brain^38^, blood vessels^39^, gastrointestinal tract^40^, pancreas^41^, and muscle^42^. However, there is little information on the role of 5-HTR in LG function. Among the seven subfamilies of 5-HTR, 5-HT3R is a ligand-gated ion channel^43^ that differs from all other 5-HTR, the actions of which are regulated via G-protein^32^. 5-HT3R has the ability to induce [Ca^2+^]i mobilization through extracellular Ca^2+^ entry by way of non-selective cation channel activation^31^. We found that 5-HT3R plays a role in LG secretory functions and confirmed that extracellular Ca^2+^ entry participates in [Ca^2+^]i mobilization stimulated by 5-HT. M3R, located on the basolateral membrane of LG^44^, and mediates a well-established pathway in tear fluid secretion in the LG. In contrast to 5-HT3R, this receptor modulates [Ca^2+^]i from intracellular Ca^2+^ stores promoted by inositol 1,4,5-trisphosphate^45^. Generally, simultaneous activation of two different receptors and their signaling pathways can lead to cooperative outcomes if the two receptors use different signaling pathways^46^. We found that the simultaneous stimulation of 5-HT3R with M3R on LG cooperatively amplified [Ca^2+^]i mobilization.

In humans, serotonin is normally present in the circulation at a level of approximately 100 to 200 ng/mL^47^. Therefore, the LG is expected to be maintained under a condition of continuous 5-HT stimulation. In parallel, a neuronal parasympathetic signal transmitted from the ocular surface sensory nerves elicited by transient spontaneous blinking, continuously stimulates the LG via projected parasympathetic nerves^12^. These, taken together with our findings of the interaction between 5-HT3aR and M3R, suggest that the [Ca^2+^]i of LG acinar cells could have been maintained at a necessary level by the simultaneous actions of blood 5-HT and the neural parasympathetic pathway, leading to LG homeostasis under physiological conditions.

We observed that a decrease in blood 5-HT correlated with loss of the ability to secrete tears, LG atrophy, and autophagy. Because these alterations were induced or blocked by treatment with 5-HT3R antagonists or agonists, respectively, the mechanisms underlying LG atrophy induced by decreased blood 5-HT can be explained by a 5-HT3R-mediated process. Autophagy is a fundamental cellular homeostatic mechanism in which cells catabolize and recycle their cytoplasm for survival^24^. It is triggered in response to radical environmental changes such as nutritional deficits^28^. In the present study, activation of LG autophagy induced by a decreased blood serotonin level was accompanied by a decrease in ATP content and increase in reactive oxygen leakage from the LG, suggesting mitochondrial dysfunction in the LG. In addition, the LG mTOR pathway, a negative integrator of signals associated with nutritional status, was inhibited as a response to decreased blood serotonin level, suggesting that the change in the energetic state of the LG was associated with blood serotonin level. Intracellular Ca^2+^ plays a role in maintaining cellular energy homeostasis, particularly mitochondrial biogenesis^48^, ATP production^49^, and secretory functions^50^. In addition, an increase in [Ca^2+^]i activates the phosphorylation of mTOR^51^, leading to attenuation of the autophagy pathway^26^. Tear flow is generated by a combined transepithelial osmotic gradient elicited by the activation of multiple water channels and ion transporters, and protein release by an intracellular vesicle-mediated exocytosis process in LG acinar cells^12^. These secretory processes require ATP as an energy source^52, 53^. Taken together with the results showing that LG [Ca^2+^]i was modulated through 5-HT3aR, our observations raise the possibility that blood 5-HT acts as an essential signal for maintaining an appropriate energy status for LG secretory function.

A previous study of the secretagogue action of 5-HT on LG demonstrated that protein secretion from acinar cells was stimulated by 5-HT originating from LG mast cell degranulation^54^, although the role of blood 5-HT on LG function remains to be defined.

**Figure 7:**
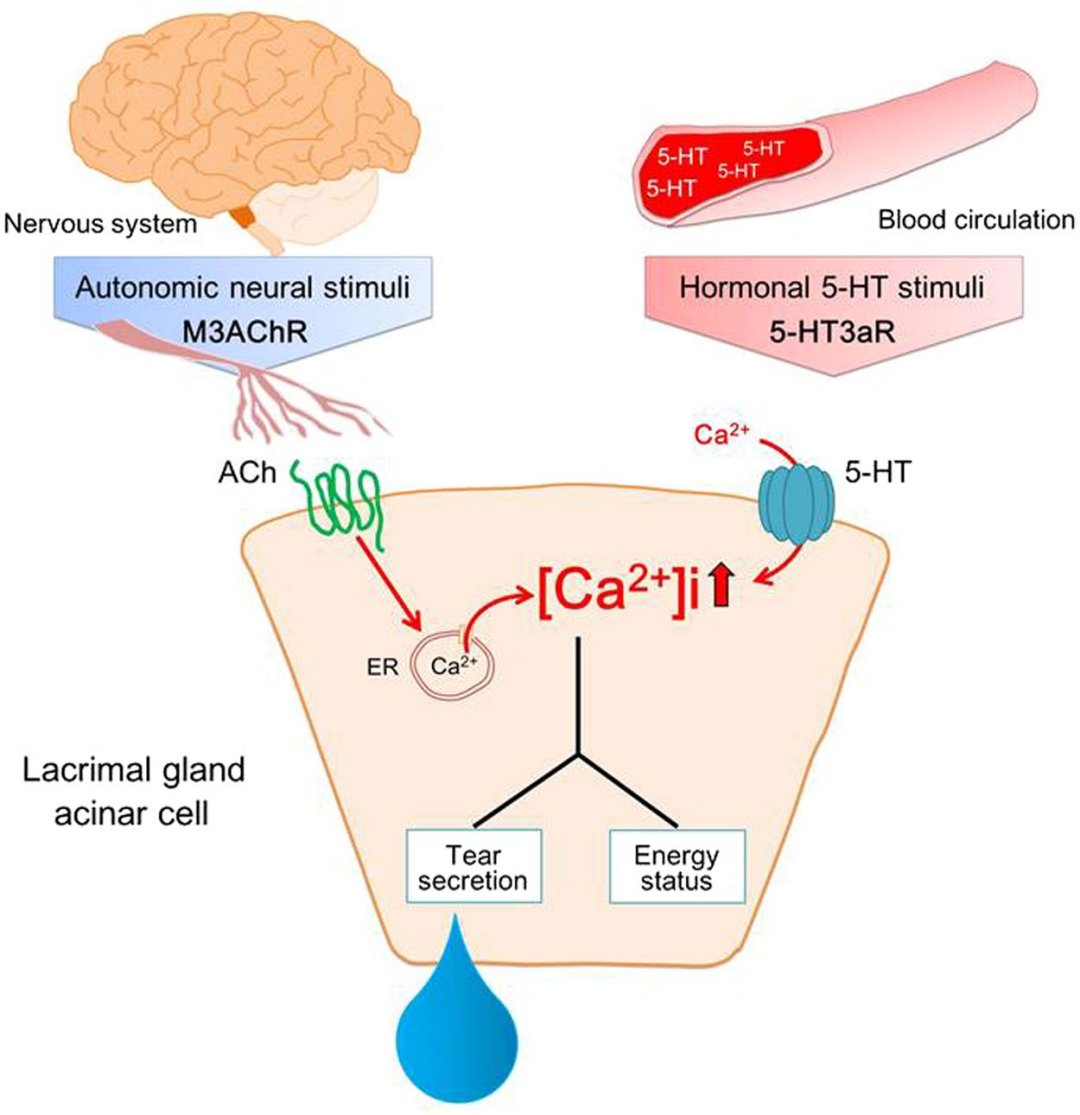
Outline of hormonal regulation of tear secretion by serotonin. 5-HT3aR, a ligand-gated ion channel that induces intracellular calcium ([Ca^2+^]i) mobilization through entry of extracellular Ca^2+^ into the cell, is located in the basolateral lacrimal gland (LG). [Ca^2+^]i in the LG acinar cells, which are responsible for maintaining aqueous tear secretion and cellular energy, is regulated by the interaction between 5-HT3aR and m3AChR activity via blood 5-HT and neuronal parasympathetic stimulation, respectively. Tear secretion is maintained at an optimum level by the simultaneous action of blood 5-HT and the neural parasympathetic pathway. Abbreviations: ACh, acetylcholine; ER, endoplasmic reticulum; m3AChR, M3 muscarinic acetylcholine receptor; 5-HT, serotonin; 5-HT3aR, serotonin type 3a receptor.

Our results show that not only parasympathetic innervation, but also physiological levels of blood 5-HT, are essential for maintaining constant tear secretion. We also demonstrated that 5-HT3R expressed in the LG participates in this function by modulating acinar cell [Ca^2+^]i. Up until the present, it was believed that parasympathetic innervation predominantly regulated LG secretory function^12^. Our findings, for the first time to our knowledge, raise the possibility that hormonal regulation involving 5-HT plays a crucial role in LG secretory function. 5-HT regulates tear secretion and LG morphology via the modulation of [Ca^2+^]i by cooperating with the autonomic nervous system (Fig. 7).

Dry eye syndrome is characterized as impairment in the status of the tear film, which results in ocular discomfort and visual impairment affecting quality of life^55^. The rate of dry eye syndrome is markedly increasing in industrialized countries, although the etiology of this condition is multifactorial and largely remains unknown^20^. There are few reports demonstrating the relationship between 5-HT and dry eye. In one cross-sectional study, tear serotonin concentration positively correlated with symptoms and signs of dry eye^56^. In addition, 5HT receptors, which play a role in pain sensation^57^, have been reported to be expressed in human conjunctiva^58^. These findings indicate the possible involvement of tear 5-HT levels on ocular surface nociperception and sensory sensitivity in dry eye. However, hormonal effect of blood 5-HT on LG tear secretory function has not been reported.

Aging is a proven risk factor of dry eye syndrome^59^. Oxidative damage governing the rate of aging is believed to play a role in pathogenesis^59^. Peripheral 5-HT varies under physiological conditions^60, 61^ and decreases with aging^47^. Together with our results showing that a decrease in the 5-HT level generated ROS production from the LG, our data showing that the blood 5-HT level correlated with LG tear secretory function suggest that an alteration of peripheral 5-HT levels potentially contributes to the pathophysiology of aging-associated dry eye syndrome. Further investigation of the clinical relevance of 5-HT in dry eye syndrome and other hormonal effects on LG secretory function will advance our understanding of both the pathogenesis of dry eye syndrome and therapeutic interventions for this condition.

## Materials and Methods

### Animals

Female C57BL/6 mice and Sprague-Dawley rats (CLEA Japan, Inc. Tokyo, Japan), at an age of 8 weeks, were used in this study (n = 140 for mice and n = 20 for rats). The care and handling of the animals were performed in accordance with the Association of Research and Vision in Ophthalmology (ARVO) statement for the Use of Animals in Ophthalmic and Vision Research. All of the experimental protocols were approved by the Ethics Committee on Animal Research of the Keio University School of Medicine (Approval No. 12111-1). Animals were quarantined and acclimatized for 1 week prior to the experiments under the following general conditions: room temperature of 23 ± 2 °C, relative humidity of 60 ± 10%, and alternating 12-hour light-dark cycle (8 AM to 8 PM), with free access to food and water. Mice were used to evaluate tear secretion *in vivo* and LG morphology. Rat-isolated LG were used for *in vitro* LG intracellular calcium ion concentration ([Ca^2+^]i) measurement. A mouse and rat were used for LG gene expression analysis by quantitative PCR and/or *in situ* hybridization (ISH).

### Tryptophan (Trp)-free diet

Dietary composition was based on a standard AIN93G diet (Oriental Yeast Co., Ltd. Tokyo, Japan). AIN93G without Trp was used as a Trp-free diet (Oriental Yeast). A standard diet was given during the acclimatization period, and then changed to a Trp-free diet for 7 days. The mice continuously fed a standard diet were used as the control group. LG secretory functions were evaluated during 7 days of Trp-free diet feeding. Whole LG was used throughout the study to perform the biochemical and histological experiments. For each experiment, 5 to 6 mice were used from each group. Blood samples were collected from the abdominal aorta and 5-HT concentration was determined using high-performance liquid chromatography (HPLC) (SRL Inc., Tokyo, Japan).

### Measurement of tear secretion

Tear secretion was measured using a modified Schirmer test^62^. A phenol red thread (Zone-Quick; Showa Yakuhin Kako, Tokyo, Japan) was placed on the temporal side of the upper eyelid margin for 15 seconds. The length of the moistened area from the edge was measured to within 0.5 mm. For the determinations of stimulated secretion by 5-HT or the parasympathetic stimulant CCH, each drug was dissolved in saline and 100 µl of each agent was injected i.p. to mice at dosages of 0.001, 0.1, and/or 1 mg/kg and 0.1 mg/kg, respectively. Tear secretion measurements were obtained 5 minutes before and every 5 minutes for 20 minutes after the injection. For the evaluation of the cooperative effect between 5-HT and CCH, 0.1 mg/kg 5-HT and 0.1 mg/kg CCH were injected simultaneously.

### Histopathological examination

Mice were euthanized with an overdose of pentobarbital sodium, and their LG were dissected. For hematoxylin and eosin (HE) staining, the LG was fixed in 10% formalin. After dehydration, LG specimens were embedded in paraffin, cross-sectioned, and stained.

For transmission electron microscopy (TEM), mice were perfused with Karnovsky’s fixative (2.5% glutaraldehyde and 2% paraformaldehyde in 0.1 M sodium cacodylate; pH 7.4) under anesthesia, and the excised LG was immersed in the fixative. One-micrometer-thick sections were stained with methylene blue, and ultrathin sections were made using a diamond knife. The ultrathin sections were collected on mesh grids, stained with uranyl acetate and lead citrate, and examined using an electron microscope (JEM-1400Plus; JEOL Ltd. Tokyo, Japan).

### Meibography of whole-mount eyelid

Mice were euthanized with an overdose of pentobarbital sodium, and their eyelids were dissected. The eyelid was fixed in 10% formalin and the specimen was then incubated overnight in 15% and then 30% sucrose in PBS. This process made the connective tissues, including palpebral tissue, transparent while keeping the lipid-rich Meibomian gland (MG) opaque.

### Periodic acid Schiff (PAS) staining

PAS staining was performed to evaluate the distribution of mucin-containing goblet cells in conjunctival tissues. Mice were euthanized with an overdose of pentobarbital sodium, and their whole eyes with lids were dissected. The specimen was fixed in 10% formalin, embedded in paraffin, and sagittal-sectioned from the middle of the eye. Each section was stained with periodic acid alcohol. After washing with deionized water, the sections were stained with Schiff reagent.

### Measurement of ATP content and reactive oxygen species (ROS) in LG

Mice were euthanized with an overdose of pentobarbital sodium. Excised whole LG was immersed in cold phosphate-buffered saline (PBS, 25 mg tissue weight/mL) and homogenized using a Mil mixer with a zirconia ball (AS ONE corporation, Osaka, Japan) for 3 minutes. Homogenized LG tissue was examined to measure ATP content and ROS generation. For measurement of ATP content, LG homogenates were incubated for 5 minutes at 98 °C. The preparations were centrifuged at 800 *g* for 3 minutes and the supernatant was used for ATP measurement. ATP content was determined using the chemiluminescence method (ATP Bioluminescence Assay Kit CLS 2, Roche Molecular Biochemicals, Mannheim, Germany). ATP calibration was performed using ATP and luciferase. ROS generation was measured using the ROS-sensitive fluorescence indicator 2′,7′-dichlorofluorescein diacetate (DCFH-DA, Molecular Probes, Eugene, OR, USA). LG homogenates were incubated with DCFH-DA for 1 hour at 37 °C. The preparations were washed 3 times with PBS by centrifugation at 800 *g* for 3 minutes. Washed cells were re-suspended in PBS, followed, and read at an excitation of 480 nm and emission of 530 nm. Each measurement was performed using a Synergy 4 plate reader (Biotek Company, Winooski, VT, USA).

### Western blot

Mice were euthanized with an overdose of pentobarbital sodium. Excised whole LG was homogenized with a Polytron homogenizer (Kinematica Polytron PT 1200E, Lucerne, Switzerland) for 1 minute in RIPA buffer (50 mM Tris/HCl pH 7.5, 150 mM NaCl, 1.0% Igepal CA-630) containing a protease inhibitor cocktail (Complete Mini, Roche Diagnostics, Basel, Switzerland). Samples were centrifuged at 4 °C at 15,000 rpm for 5 minutes and the protein concentration in each LG homogenate was determined using a DC-protein assay kit (Bio-Rad, Hercules, CA, USA). Next, the same volume of 2× Laemmli sample buffer was added as well as 5% β-mercaptoethanol. After boiling, samples were separated by polyacrylamide gel electrophoresis, transferred to PVDF membranes using a dry blotting system (V20-SDB, SCIE-PLAS, Warwickshire, UK), and incubated with a rabbit polyclonal antibody against p62 (Medical and Biological Laboratories, Aichi, Japan), microtubule-associated protein 1 light chain 3 (LC3, Novus Biologicals, Littleton, CO, USA), mammalian target of rapamycin (mTOR, rabbit polyclonal, 1:1000 dilution, Cell Signaling Technology, Tokyo, Japan), phosphor-mTOR (rabbit polyclonal, 1:1000 dilution, Cell Signaling Technology), ribosomal protein S6 (rabbit polyclonal, 1:1000 dilution, Cell Signaling Technology), phosphor-S6 (rabbit polyclonal, 1:1000 dilution, Cell Signaling Technology), manganese superoxide dismutase (SOD2, rabbit polyclonal, 1:200 dilution, Santa Cruz Biotechnology, Santa Cruz, CA, USA), or mouse polyclonal antibody against β-actin (1:2000 dilution, Sigma-Aldrich Japan, Tokyo, Japan). Goat anti-rabbit antibodies or goat anti-mouse antibodies conjugated to alkaline phosphatase (1:5000 dilution, Promega, Tokyo, Japan) and Nitroblue tetrazolium chloride/5-bromo-4-chloro-3-indolylphosphate p-toluidine salt (NBT/BCIP) compounds (Roche, Switzerland) were used to visualize immunolabeled proteins. Band intensities were quantified using ImageJ software (National Institutes of Health, Bethesda, MD, USA).

### Administration of 5-HTR antagonist or agonist

Way-100635 (5-HT1aR antagonist, Sigma-Aldrich), ketanserin (5-HT2aR antagonist, Sigma-Aldrich), ondansetron (5-HT3R antagonist, LKT Laboratories Inc., Saint Paul, MN, USA), and SB269970 (5-HT7R antagonist, Sigma-Aldrich) were used as 5-HTR antagonists, and SR57227A (Sigma-Aldrich) was used as a 5-HT3R agonist. Saline was used as a vehicle control. The infusion of each agent at a uniform rate was achieved using an osmotic pump (0.24 µL/h for 14 days; Alzet, Cupertino, CA, USA) implanted subcutaneously in the ventral part of the mice under inhalation anesthesia by isoflurane. The dosage of each antagonist and SR57227A was designed to be continuously administered at 120 and 72 µg/kg/day, respectively. Each antagonist was infused into mice kept on a standard diet, and SR57227A was infused 1 day before changing from a standard to a Trp-free diet. Each antagonist was infused for 7 days and SR57227A was infused for 8 days.

For determination of the inhibitory effect of ondansetron on 5-HT-stimulated secretion, 1 mg/kg 5-HT and 10 mg/kg of ondansetron were simultaneously injected i.p. The agonist SR57227A was injected at a dosage of 1 mg/kg i.p. Each experiment was performed after 2 days of feeding a Trp-free diet.

### Intracellular calcium ion concentration ([Ca^2+^]i) measurements

Rats were euthanized with an overdose of pentobarbital sodium. LG isolated from normal rats was digested by collagenase type 3 (Worthington, Lakewood, NJ, USA) and filtered through a 100-µm nylon mesh (Cell Strainer, BD Biosciences, Shizuoka, Japan) to isolate LG acinar cells. Acinar cells were loaded with fura-2/AM (Invitrogen, Gaithersburg, MD, USA), a fluorescent Ca^2+^ indicator, with 0.01% Pluronic F-127 (Invitrogen, USA) before measuring [Ca^2+^]i using the dual-wavelength microfluorescence technique, as described previously^63^.

Acinar cells were continuously perfused with a saline solution (140 mM NaCl, 5 mM KCl, 10 mM CaCl_2_, 1 mM MgCl_2_, 10 mM HEPES, 10 mM dextrose [pH 7.4]). Solutions of 5-HT (0.1, 1, and 10 µM) and SR57227A (0.1, 1, and 10 µM) were diluted to the desired concentrations with a saline solution and used as stimulants. Ondansetron (10 µM) was prepared in saline solution, and a Ca^2+^-free solution was produced by omitting CaCl_2_. For evaluation of the effect of ondansetron and a Ca^2+^-free solution on 5-HT–mobilized [Ca^2+^]i, each solution was applied to acinar cells 2 minutes before the application of 5-HT (1 µM) to each solution. For evaluation of the simultaneous effect of 5-HT and ACh on [Ca^2+^]i mobilization, ACh (0.1, 1, 10, 100, and 1000 µM) was applied with 5-HT (1 µM) to acinar cells. For each experiment, 33 to 49 acinar cells were used in each group.

### *In situ* hybridization (ISH)

Normal rats were anesthetized with pentobarbital (80 mg/kg) and perfused with 4% paraformaldehyde phosphate-buffer; excised whole LG was postfixed in the same fixative overnight. Fixed LG were cryoprotected in diethylpyrocarbonate-treated PBS containing 20% sucrose, embedded in O.C.T. compound, and then cryosectioned at 7 µm thickness. Sectioned specimens were treated with 40 µg/ml proteinase K for 30 minutes at room temperature (25 ± 5 °C). After acetylation with 0.25% acetic anhydride in 1% triethanolamine solution for 10 minutes, pre-hybridization was carried out for 2 hours at 63 °C in a hybridization buffer consisting of 50% formamide, 5× saline sodium citrate (SSC) buffer, 5× Denhardt’s solution, and 0.4 mg/ml salmon sperm DNA. After pre-hybridization, digoxigenin (DIG)-labeled cRNA probes were hybridized to sectioned specimens at 60 °C overnight. After hybridization, specimens were washed with 5× SSC at 60 °C for 5 minutes, 2× SSC at 60 °C for 5 minutes, 0.2× SSC/50% formamide at 60 °C for 30 minutes, and 0.2× SSC at room temperature for 10 minutes. After a stringent wash, specimens were incubated with 1% blocking regent (Roche) for 60 minutes, followed by incubation with an alkaline phosphate-conjugated anti-DIG antibody (1:5000 dilution, Roche) for 90 minutes at room temperature. NBT/BCIP compounds (Roche) were used for color development, and nuclear fast red (Vector Lab, Burlingame, CA, USA) was used for counterstaining. The sequences of the probes for 5-HTR subtypes were previously described^38^. A full-length M3R cDNA (NCBI accession number: NM_033269.4) was used for M3R cRNA probe.

### Quantitative real-time PCR

Total RNA was isolated from normal rat LG using an RNA extraction reagent (ISOGEN; Nippon Gene, Tokyo, Japan), according to the manufacturer’s instructions. RNA was used for reverse transcription, and then cDNA synthesis was performed using the ReverTra Ace qPCR RT kit (TOYOBO, Osaka, Japan). SYBR Green-based quantitative real-time PCR was performed using the Step One Plus System (Applied Biosystems, Framingham, MA, USA). Levels of mRNA were evaluated using the ΔΔCt method and normalized to the level of glyceraldehyde 3-phosphoate dehydrogenase (GAPDH). Each PCR amplification was performed using a specific primer set. The primer sequences were as follows: 5-HT3aR (sense 5′-TCTTGCTGCCCAGTATCTTCCTCA-3′, antisense 5′-TTATGCACCAGCCGCACAATGAAG-3′), 5-HT3bR (sense 5′-TGAGGCCACCATGTCTACC-3′, antisense 5′-TCATCGTTCCAAACCTCTCG-3′), M3R (sense 5′-GTGCCATCTTGCTAGCCTTC-3′, antisense 5′-TCACACTGGCACAAGAGGAG-3′), GAPDH (sense 5′-GATGCTGGTGCTGAGTATGTCG-3′, antisense 5′-GTGGTGCAGGATGCATTGCTGA-3′).

### Parasympathetic post-ganglionic denervation (PGD)

Mice were placed in a prone position, and the skin on the right temporal side of the head was incised while the mice were anesthetized using an i.p. injection of pentobarbital (80 mg/kg). The post-ganglionic nerve bundle was detached from the blood vessels at the caudal root site of the ventral surface of the LG and denervated under a stereomicroscope.

To investigate the interaction between neuronal parasympathetic and blood 5-HT stimuli in the LG, PGD surgery was performed in the mice with feeding of a Trp-free diet or a standard diet for 3 days. After 1 day of PGD surgery, the LG morphology was evaluated. For each experiment, 5 mice were used in each group.

### Statistical analysis

Student’s *t*-test was used for comparisons between two groups and Dunnett’s test was used for multiple comparisons. Differences were considered significant if the *P*-value was 0.05 or less.

## Electronic supplementary material

Supplementary information
