# Supplementary material for: Serotonin hormonally regulates lacrimal gland secretory function via the serotonin type 3a receptor
Source: Sci Rep. 2017 Jul 31;7:6965. doi: 10.1038/s41598-017-06022-4 (PMC5537296; doi:10.1038/s41598-017-06022-4)

Title

Serotonin hormonally regulates lacrimal gland secretory function via serotonin type 3a receptor

Author names

Toshihiro Imada[1], Shigeru Nakamura[1*], Ryuji Hisamura[1], Yusuke Izuta[1], Kai Jin[1], Masataka Ito[2], Naoki Kitamura[3], Kenji F. Tanaka[4], Masaru Mimura[4], Izumi Shibuya[3], Kazuo Tsubota[1*]

1) Department of Ophthalmology, Keio University School of Medicine, Tokyo, Japan

2) Department of Ophthalmology, National Defense Medical College, Saitama, Japan

3) Department of Veterinary Physiology, Faculty of Agriculture, Tottori University, Tottori, Japan

4) Department of Neuropsychiatry, Keio University School of Medicine, Tokyo, Japan

Contact information:

*Shigeru Nakamura

Department of Ophthalmology

Keio University School of Medicine

35 Shinanomachi, Shinjyuku-ku, Tokyo 160-8582, Japan

Tel: +81-3-5363-3972

Fax: +81-3-5363-3974
E-mail: vdtwork@gmail.com

*Kazuo Tsubota
Department of Ophthalmology
Keio University School of Medicine
35 Shinanomachi, Shinjyuku-ku, Tokyo 160-8582, Japan
Tel: +81-3-5363-3972
Fax: +81-3-5363-3974
E-mail: tsubota@z3.keio.jp

Supplementary Information

Supplementary Materials and Methods

Protein secretion from lacrimal gland (LG) acinar cells

Rats were euthanized with an overdose of pentobarbital sodium. The excised LG was digested by collagenase type 3 (Worthington, USA) and incubated in saline solution (140 mM NaCl, 5 mM KCl, 2 mM $CaCl_2$, 1 mM $MgCl_2$, 10 mM HEPES, 10 mM dextrose [pH 7.4]) with 5-hydroxytryptamine (5-HT) for 60 minutes at 37°C. The preparations were centrifuged at 800 g for 3 minutes and the protein concentration in the supernatant measured using Bradford reagent (Sigma-Aldrich, USA), with bovine serum albumin (BSA) as the standard. The protein secretion rate was calculated as a percentage of that before stimulation. Solutions of 5-HT (0.1, 1, 10, 100, and 1,000 µM) were diluted to the desired concentrations with saline solution and used as stimulants. Ondansetron (10 µM) was appropriately prepared in saline solution before use. For each experiment, six LGs were used in each group.

***In situ* hybridization (ISH) in mouse lacrimal gland**

Mice were euthanized with an overdose of pentobarbital sodium and their LGs rapidly dissected. *In situ* hybridization was performed as described under Methods.

Supplementary Figure Legends

Supplementary Figure 1. Effect of Trp-free diet on tear secretion in male mice

Mice were fed either a standard (control group) or a Trp-free diet (Trp-free group) for 7 days after the acclimatization period. (A) Changes in tear secretion. Each measurement was performed during 7 days of feeding with either a standard or a Trp-free diet. Atrophic alterations in the LG size (B) and weight (C) were recorded on day 7 after the switch to the Trp-free diet. The dotted line indicates LG (B, the scale bar is 2 mm). Each panel shows the control (upper) and Trp-free diet (lower) groups. Open and solid symbols indicate the control and Trp-free groups, respectively (A, C). Data represent the mean ± SD, n=5 mice. ***P < 0.001 versus control, ### P < 0.001 versus day 0.

Supplementary Figure 2. Recovery of tear secretion and lacrimal gland weight after reverse switch from Trp-free to standard diet

Mice were given a Trp-free diet for 7 days followed by a reverse switch to the standard diet. (A) Recovery of tear secretion (n=7 mice). (B) Recovery of LG weight (n=8-10 LG). Right photographs show the typical gross appearance of LG after 3 (upper) and 7 (lower) days of reverse switch to the standard diet from Trp-free diet (the scale bar is 2 mm). The dotted line indicates LG. All data represent the mean ± SD, **P < 0.01,

***P < 0.001 versus day 0.

Supplementary Figure 3. Effect of Trp-free diet on peripheral tissue weight

(A) Changing ratio of tissue weight on day 7 after the switch to Trp-free diet. Data show the ratio of tissue weight of the Trp-free diet group to that of the standard diet group (control, red dotted line). Mean ± SD, n = 4-8. *P < 0.05, ***P < 0.001 versus corresponding control, †††P < 0.001 versus liver, §§§P < 0.001 versus submandibular gland. (B) Alteration in the size of the liver (upper) and submandibular gland (lower) at day 7 after the switch to Trp-free diet. Each photograph shows the control (left) and Trp-free diet (right) groups. The scale bar is 5 mm.

Supplementary Figure 4. Effects of ondansetron infusion on lacrimal gland autophagy

Mice were infused with either saline (vehicle) or ondansetron for 7 days via an osmotic pump. (A) Alteration in LG size. The dotted line indicates LG (the scale bar is 2 mm). (B) Changes in the expression of autophagy-related proteins. Data show the representative western blot images and quantitative analysis (bar chart) at day 7 after systemic infusion of either the vehicle or ondansetron. Open and solid columns indicate

the vehicle and ondansetron groups, respectively. All data represent the mean ± SD, n=5 LG. *** $P < 0.001$ versus corresponding vehicle.

Supplementary Figure 5. Stimulated tear secretion by serotonin and SR57227A in mice with blood serotonin concentration under the normal level

Mice were intraperitoneally injected with either saline (vehicle, open circle), 5-HT (closed circle), or SR57227A (closed triangle). Tear secretion was measured 5 minutes before and every 5 minutes for 30 minutes after injection. Data represent the mean ± SD, n=5 mice. *$P < 0.05$ versus 0 min.

Supplementary Figure 6. Protein release from isolated rat lacrimal gland acinar cells after stimulation with 5-HT

Exocytotic release of protein was determined as the amount of total protein release from LG acinar cells isolated from normal rat. (A) Total protein release from LG acinar cells induced by 5-HT stimulation. The LG was stimulated with 5-HT at dosage ranging from 0.1 to 1,000 μM. (B) Inhibitory effects of ondansetron (10 μM) on total protein release from LG acinar cells by 5-HT (100 μM). All data represent the mean ± SD, n=5 LG. ***$P < 0.001$ versus control.

Supplementary Figure 7. In situ hybridization analysis of 5-HTR mRNA expression in mouse lacrimal gland

The mRNA expression of the 5-HTR subtype was determined in normal mouse LG. In situ hybridizations are shown for the indicated 5-HTR and M3R probes. Each panel shows LG acinar cells. Nuclear fast red was used as the counter stain. The scale bar is 10 µm.

Supplementary Figure 8. Morphological changes in ductal cells of the lacrimal gland

Morphological changes in LG ductal cells by 5-HT3 receptor antagonist/agonist infusion (A), and parasympathetic post-ganglionic denervation (PGD) combined with a Trp-free diet (B). Ondansetron, a 5-HT3 receptor antagonist, was infused into mice for 7 days via an osmotic pump. SR57227A, a 5-HT3 receptor agonist, was infused into mice under conditions in which blood 5-HT levels were decreased by a Trp-free diet. PGD was performed 3 days after feeding a Trp-free diet or a standard diet. Morphological changes in LG were evaluated 1 day after PGD. Saline infusion was used as vehicle control. Scale bar = 10 µm.

Supplementary Figure 1

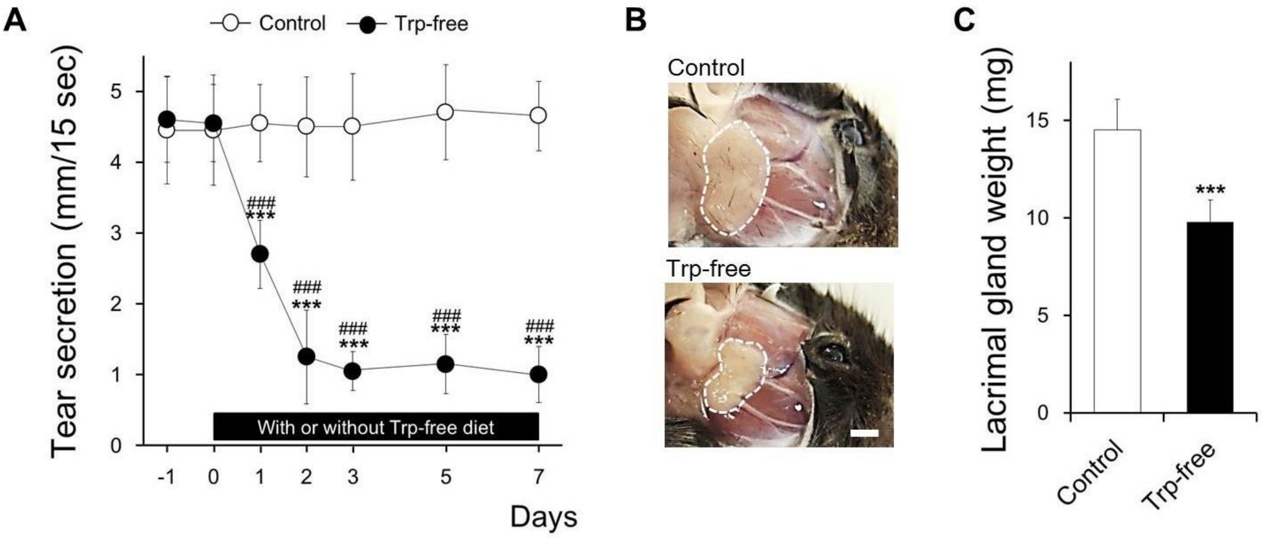

Supplementary Figure 2

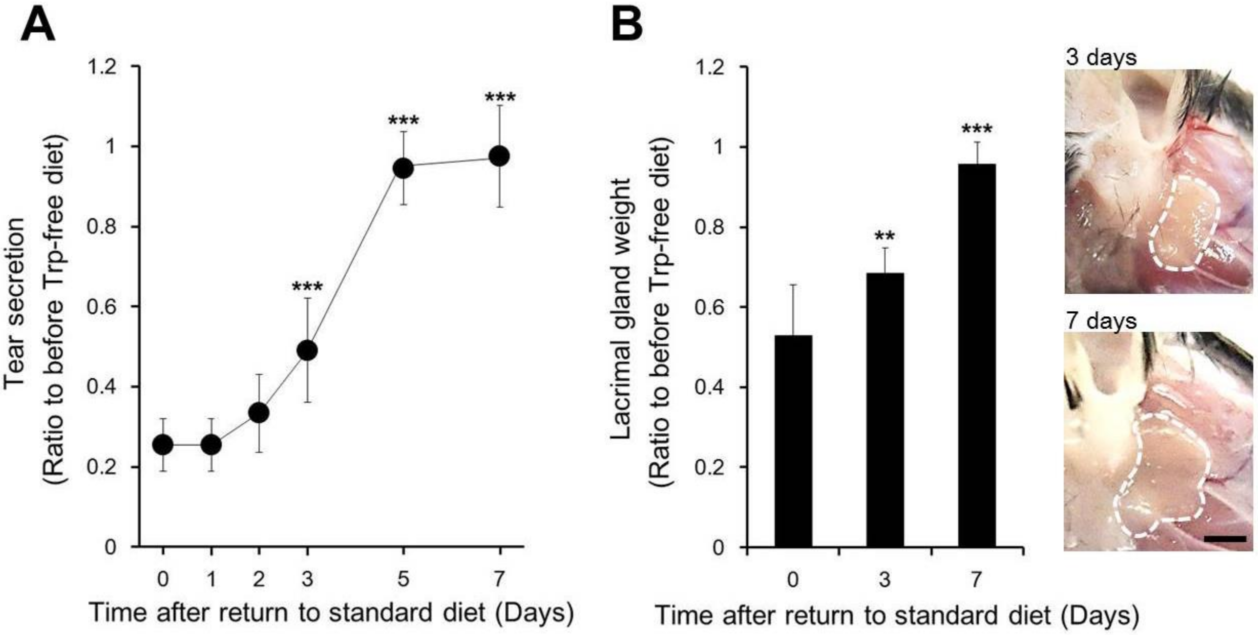

Supplementary Figure 3

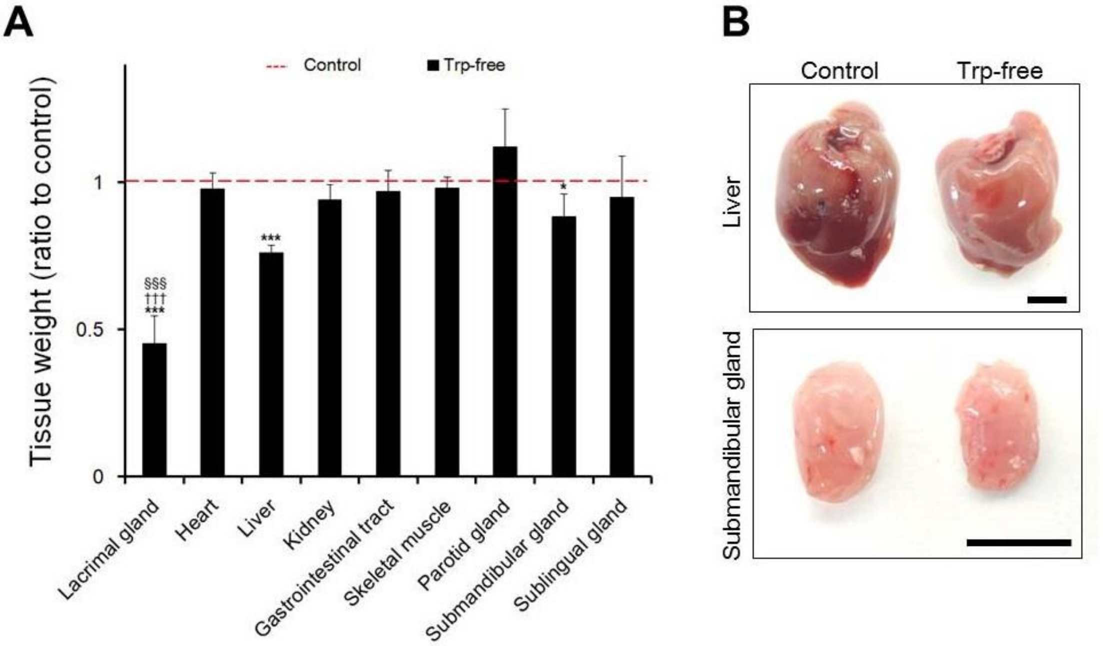

Supplementary Figure 4

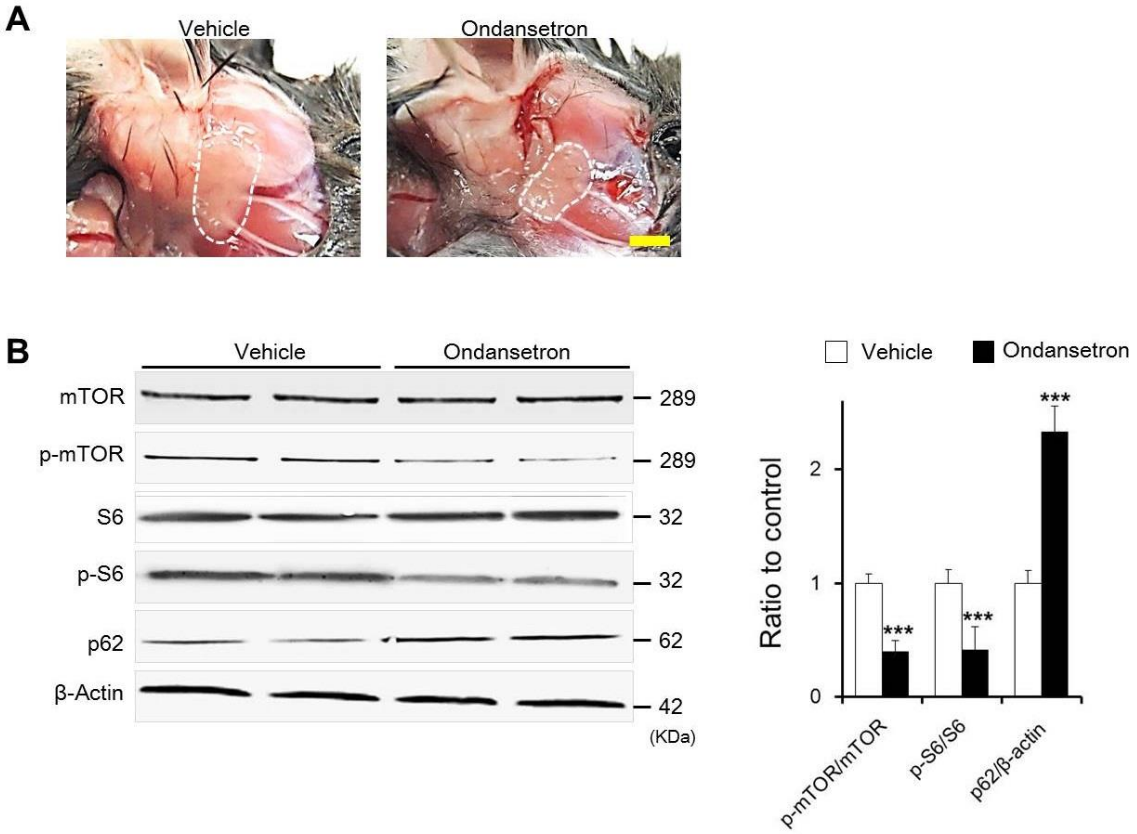

Supplementary Figure 5

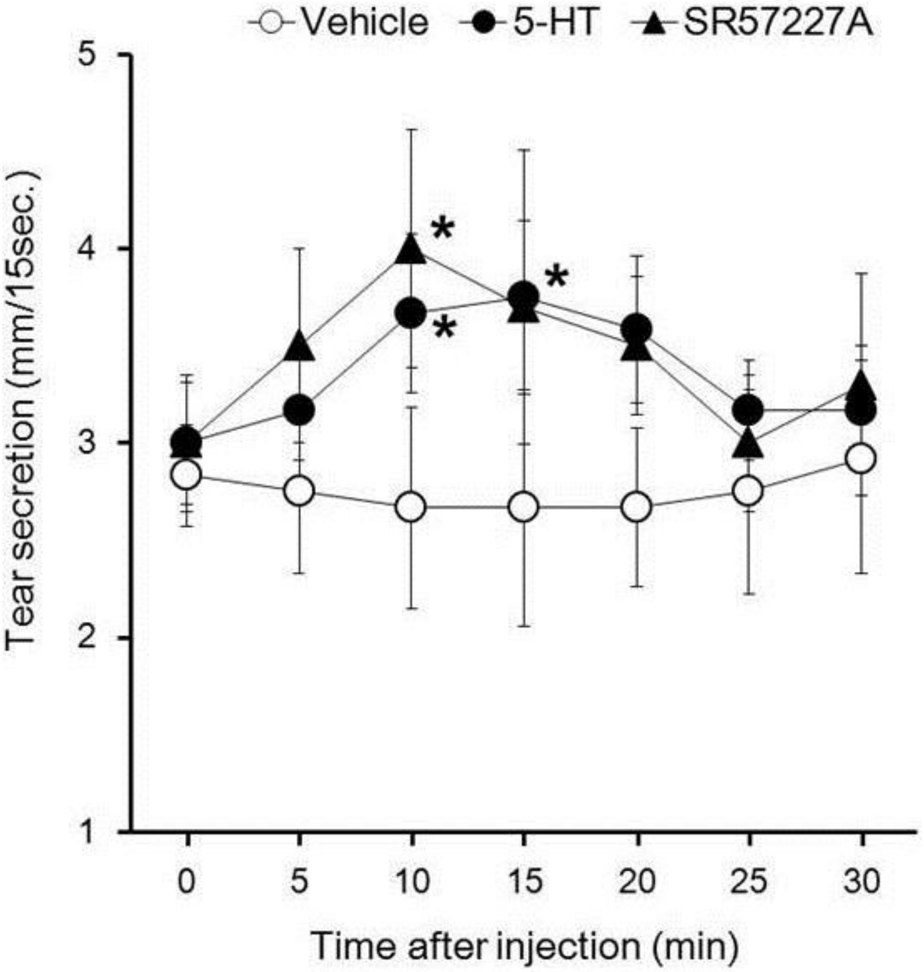

Supplementary Figure 6

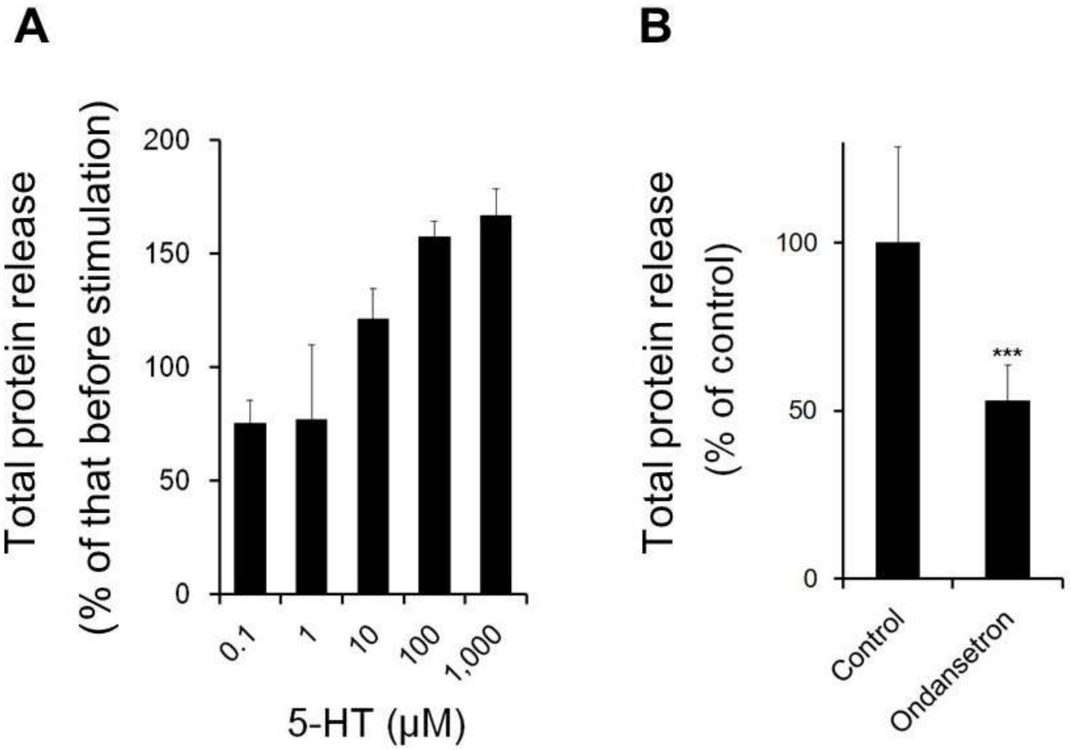

1 **Supplementary Figure 7**

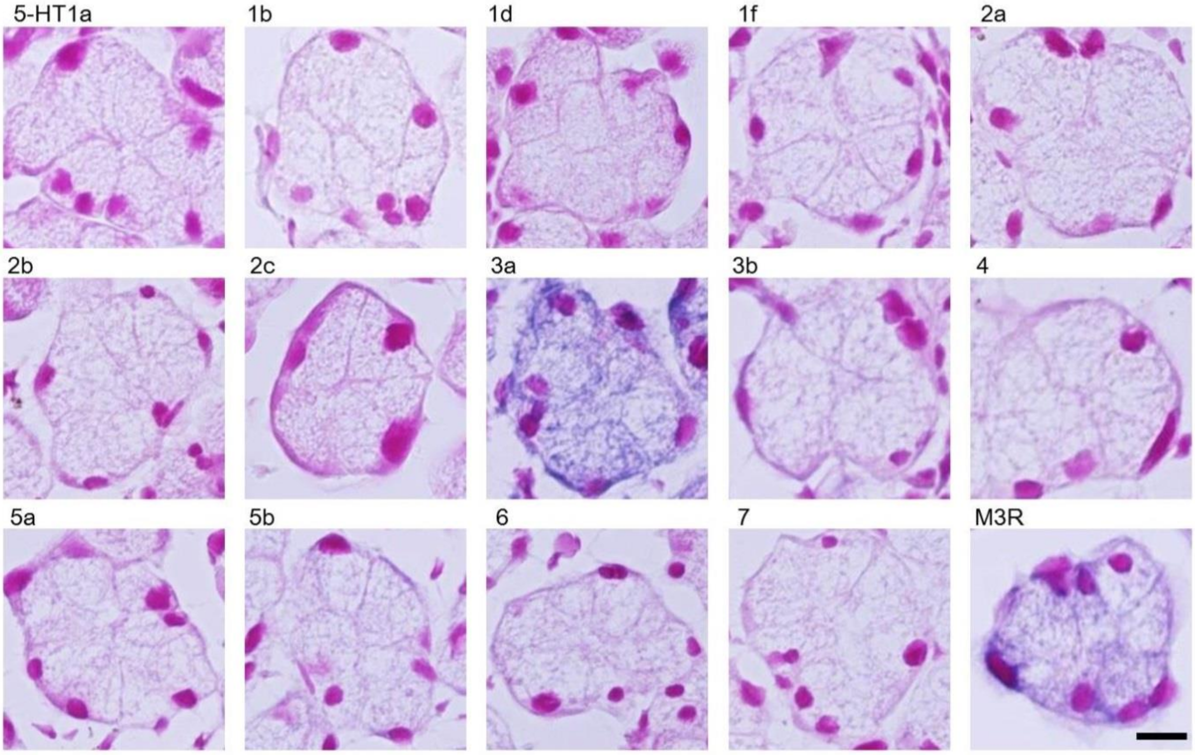

2

3

Supplementary Figure 8

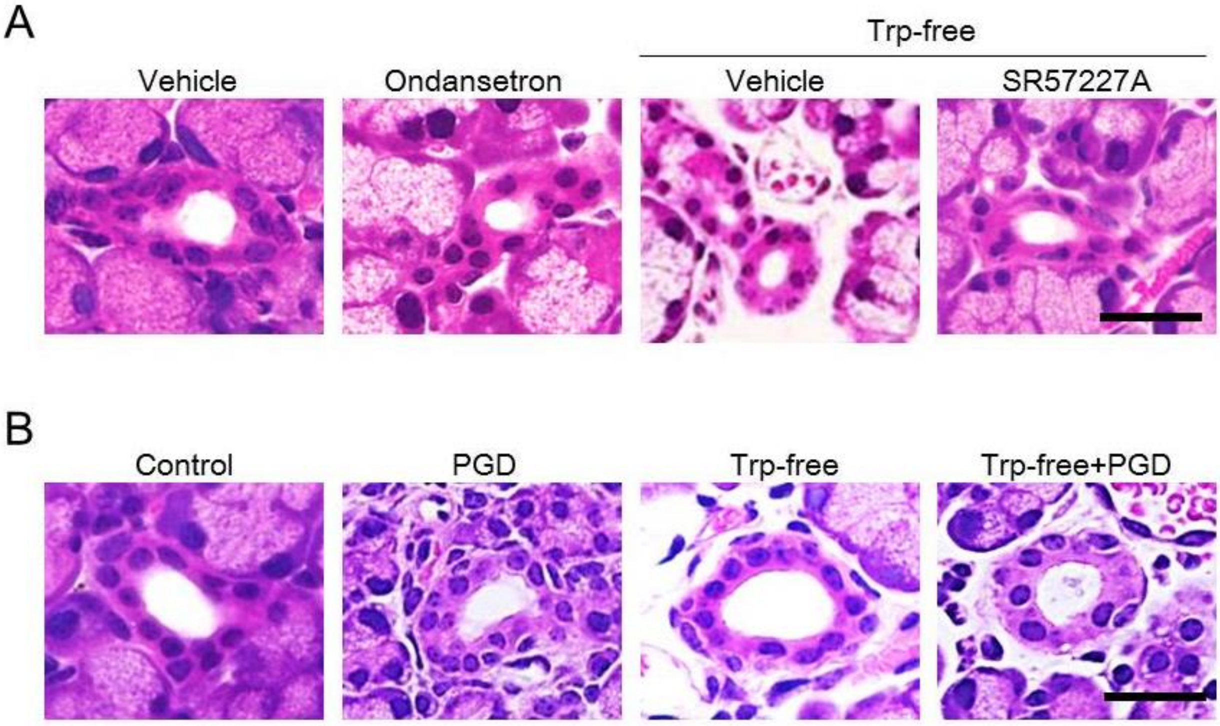

Supplement: Supplementary file 1 — Supplementary information [file 41598_2017_6022_MOESM1_ESM.pdf]
